# Impact of mutational profiles on response of primary oestrogen receptor-positive breast cancers to oestrogen deprivation

**DOI:** 10.1038/ncomms13294

**Published:** 2016-11-09

**Authors:** Pascal Gellert, Corrinne V. Segal, Qiong Gao, Elena López-Knowles, Lesley-Ann Martin, Andrew Dodson, Tiandao Li, Christopher A. Miller, Charles Lu, Elaine R. Mardis, Alexa Gillman, James Morden, Manuela Graf, Kally Sidhu, Abigail Evans, Michael Shere, Christopher Holcombe, Stuart A. McIntosh, Nigel Bundred, Anthony Skene, William Maxwell, John Robertson, Judith M. Bliss, Ian Smith, Mitch Dowsett, POETIC Trial Management Group and Trialists, Stephen Johnston, Radha Todd, Kieran Horgan, Stephen Chan, Simon D. H. Holt, Marina Parton, Ian Laidlaw, Jayant S. Vaidya, Tracey Irvine, Fiona Hoar, Ilyas Khattak, Ashutosh Kothari, Lucy Brazil, Nicholas Gallegos, Duncan Wheatley, Tayo Johnson, Geoffrey Sparrow, Serena Ledwidge, Caroline Mortimer, Marcus Ornstein, Douglas Ferguson, Douglas Adamson, Ramsey Cutress, Richard Johnson, Clare Crowley, Zoe Winters, Hisham Hamed, Russell Burcombe, Susan Cleator, Muireann Kelleher, Jonathan Roberts, Sarah Vesty, Maher Hadaki, Mary Quigley, Julie Doughty, Siobhan Laws, Seema Seetharam, Amanda Thorne, Peter Donnelly

**Affiliations:** 1 Breast Cancer Now Research Centre at The Institute of Cancer Research, 237 Fulham Road, London SW7 3RP, UK; 2 Ralph Lauren Centre for Breast Cancer Research, Royal Marsden Hospital, Fulham Road, London SW3 6JJ, UK; 3 McDonnell Genome Institute, Washington University School of Medicine, 4444 Forest Park Boulevard, St Louis, 63108 Missouri, USA; 4 Clinical Trials and Statistics Unit at The Institute of Cancer Research, 15 Cotswold Road, Sutton SM2 5NG, UK; 5 Poole General Hospital, Longfleet Road, Dorset BH15 2JB, UK; 6 Southmead Hospital, Westbury-on-Trym, Bristol BS10 5NB, UK; 7 Royal Liverpool University Hospital, 200 London Road, Liverpool L3 9TA, UK; 8 Queen's University Belfast, University Road, Belfast BT7 1NN, UK; 9 University Hospital of South Manchester, Education and Research Centre, Southmoor Road, Manchester M23 9LT, UK; 10 Royal Bournemouth Hospital, Castle Ln E, Bournemouth BH7 7DW, UK; 11 Withybush General Hospital, Fishguard Road, Haverfordwest SA61 2PZ, UK; 12 University of Nottingham, Derby Road, Nottingham NG7 2UH, UK; 13 Royal Marsden Hospital, Downs Road, Sutton SM2 5PT, UK; 14 Royal Victoria Infirmary, Queen Victoria Road, Newcastle upon Tyne NE1 4LP, UK; 15 St James's University Hospital, Beckett Street, Leeds LS9 7TF, UK; 16 Nottingham City Hospital, Hucknall Road, Nottingham NG5 1PB, UK; 17 Prince Phillip Hospital, Dafen, Llanelli SA14 8QF, UK; 18 Frimley Park Hospital, Portsmouth Road, Frimley GU16 7UJ, UK; 19 Whittington Hospital NHS Trust, Highgate Hill, London N19 5NF, UK; 20 Royal Surrey County Hospital, Egerton Road, Guildford GU2 7XX, UK; 21 City Hospital, Dudley Road, Birmingham B18 7QH, UK; 22 Ysbyty Gwynedd, Penrhosgarnedd, Bangor LL57 2PW, UK; 23 Guy's Hospital, Great Maze Pond, London SE1 9RT, UK; 24 Weston General Hospital, Grange Road, Uphill, Weston-Super-Mare BS23 4TQ, UK; 25 Royal Cornwall Hospital, London Road, Treliske, Truro TR1 3LJ, UK; 26 St Peter's Hospital, Guildford Road, Chertsey KT16 0PZ, UK; 27 Yeovil District Hospital, Higher Kingston, Yeovil BA21 4AT, UK; 28 St Bartholomew's Hospital, West Smithfield, London EC1A 7BE, UK; 29 Ipswich Hospital, Heath Road, Ipswich IP4 5PD, UK; 30 Homerton University Hospital, Homerton Row, London E9 6SR, UK; 31 Royal Devon and Exeter Hospital, Barrack Road, Exeter EX2 5DW, UK; 32 Ninewells Hospital, Dundee DD1 9SY, UK; 33 Southampton General Hospital, Tremona Road, Southampton SO16 6YD, UK; 34 Neath Port Talbot Hospital, Baglan Way, Port Talbot SA12 7BX, UK; 35 Salisbury District Hospital, Odstock Road, Salisbury SP2 8BJ, UK; 36 Bristol Royal Infirmary, Marlborough Street, Bristol BS2 8HW, UK; 37 Guy's Hospital, Great Maze Pond, London SE1 9RT, UK; 38 Maidstone Hospital, Hermitage Lane, Barming, Maidstone ME16 9QQ, UK; 39 Tunbridge Wells Hospital, Tonbridge Road, Tunbridge Wells TN2 4QL, UK; 40 St Mary's Hospital, 41 Praed Street, London W2 1NY, UK; 41 Charing Cross Hospital, Fulham Palace Road, London W6 8RF, UK; 42 St George's Hospital, Blackshaw Road, London SW17 0QT, UK; 43 King's College Hospital, Denmark Hill, London SE5 9RS, UK; 44 General Hospital, Sandford Road, Cheltenham GL53 7AN, UK; 45 Maidstone Hospital, Hermitage Lane, Barming, Maidstone ME16 9QQ, UK; 46 Queen's Hospital, Rom Valley Way, Romford, Essex RM7 0AG, UK; 47 Western Infirmary, Dumbarton Road, Glasgow G11 6NT, UK; 48 Royal Hampshire County Hospital, Romsey Road, Winchester SO22 5DG, UK; 49 Darent Valley Hospital, Darenth Wood Road, Dartford DA2 8DA, UK; 50 Musgrove Park Hospital, Taunton & Somerset NHS Foundation Trust, Taunton TA1 5DA, UK; 51 Torbay District General Hospital, Newton Road, Lawes Bridge, Torquay TQ2 7AA, UK

## Abstract

Pre-surgical studies allow study of the relationship between mutations and response of oestrogen receptor-positive (ER+) breast cancer to aromatase inhibitors (AIs) but have been limited to small biopsies. Here in phase I of this study, we perform exome sequencing on baseline, surgical core-cuts and blood from 60 patients (40 AI treated, 20 controls). In poor responders (based on Ki67 change), we find significantly more somatic mutations than good responders. Subclones exclusive to baseline or surgical cores occur in ∼30% of tumours. In phase II, we combine targeted sequencing on another 28 treated patients with phase I. We find six genes frequently mutated: *PIK3CA*, *TP53*, *CDH1*, *MLL3*, *ABCA13* and *FLG* with 71% concordance between paired cores. *TP53* mutations are associated with poor response. We conclude that multiple biopsies are essential for confident mutational profiling of ER+ breast cancer and *TP53* mutations are associated with resistance to oestrogen deprivation therapy.

## Abstract (web summary)

Aromatase inhibitors are used to treat oestrogen receptor positive breast cancers but the molecular basis for the response of patients is unclear. Here, the authors use samples from an aromatase inhibitor clinical trial and show that tumours from poor responders have more mutations than good responders and also more frequently harbour p53 mutations.

## Graphical abstract

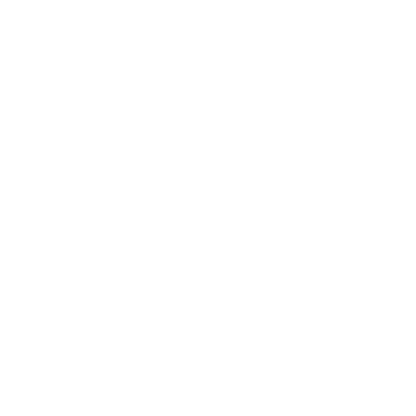

Assessment of somatic mutations is becoming increasingly important for the management of cancer patients, but molecular heterogeneity occurs across many tumours^1^. This variability is of particular interest in relation to the clonal evolution of individual malignancies, but it also poses a severe analytical challenge in terms of the degree to which the whole tumour mutational repertoire is represented by limited biopsy material.

In breast cancer, there is major interest in the use of pre-surgical studies for assessing the biological effect of therapeutic agents^2^, including the impact that the agents may have on the responsiveness of subpopulations and the emergence of subclones resistant to therapy. However, such studies inevitably depend on analyses of sequential, usually core-cut biopsies that often sample <1% of the tumour mass and may therefore provide limited representation of the tumour genotype.

Breast cancer is the most common malignancy in females in western countries and oestrogen receptor-positive (ER+) tumours contribute ∼75% of the disease^3^. Aromatase inhibitors (AIs) are the most effective agents in post-menopausal woman reducing recurrence rates in primary breast cancer patients by ∼ 50% (ref. 4). These agents inhibit aromatase throughout the body by >97% and suppress plasma oestrogen levels to undetectable levels^5^. However, these therapies are not effective in every patient. Hence, identifying the role that mutations play in *de novo* resistance to AIs is an important clinical research goal.

One large pre-surgical study, Perioperative Endocrine Therapy—Individualising Care (POETIC) trial, randomized 4,486 patients to receive 2-week non-steroidal AI or no treatment before surgery^2^. Biopsies were collected at diagnosis and at surgery to correlate molecular alterations in the tumours with their antiproliferative response to an AI. This provides the opportunity to identify DNA alterations that are of biological interest in relation to oestrogen responsiveness and of potential clinical importance in relation to AI use^6^. Like other pre-surgical studies, POETIC is potentially affected by within-tumour heterogeneity. The control group of POETIC (no pre-surgical treatment) allows us to study discrepancies between repeat biopsies from the same patients and to evaluate the molecular heterogeneity within the tumours.

In phase I of the current study, we conduct whole-exome analysis followed by capture-probe validation of baseline and surgical core-cut biopsies and of whole blood DNA. We select patients from the control group and treated patients at the extreme ends of the Ki67 response spectrum to study. On the exome-wide mutational profile, we find a significantly higher mutational load in poor responding patients indicative for multiple resistance mechanism. Over 2 weeks of treatment, we only find minor effects on the mutational profile in terms of mutational load and variant allele fractions (VAFs). In ∼30% of the tumours, we detect intra-tumoural heterogeneity with subclones exclusively to one of the core-cuts. In phase II, we perform capture-probe sequencing of baseline and surgical core-cut biopsies and whole blood DNA on additional patients. We concentrate our analysis on mutations in 77 breast cancer genes, for which the entire coding-sequence was added to the capture-panel. Through integrating the data from phase I and II, we find a reduced suppression of Ki67 within the poor responder group for *TP53*-mutated tumours and therefore a potential marker for poor response to oestrogen deprivation therapy. We show concordant detection of the mutation status of frequently mutated genes in 76% of the cases. Together with the subclonal analysis, we conclude that limited tumour material from core-cuts complicates mutational profiling of ER+ breast cancer. Multiple biopsies are required for confident mutation calling, especially for heterogeneous tumours.

## Results

### Clinical cohort

**Figure 1:**
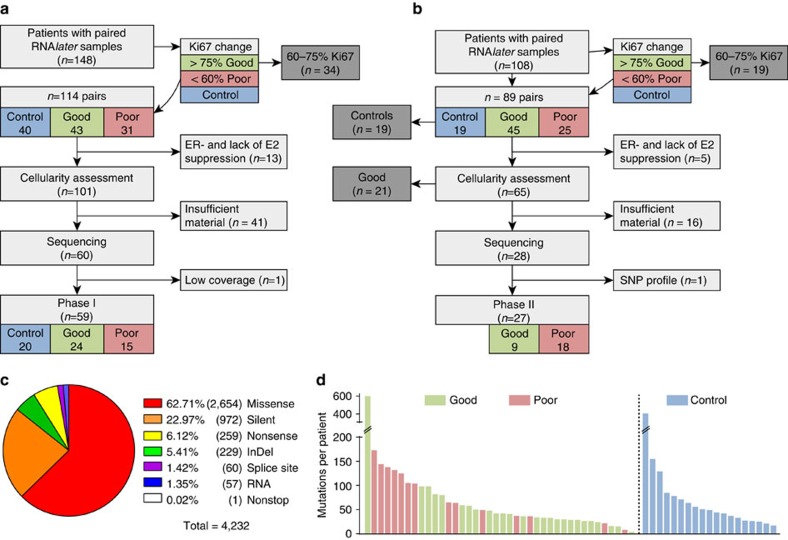
CONSORT diagram and mutational landscape. Samples were selected in two phases using the same quality criteria. Samples in phase I (**a**) underwent whole-exome sequencing (WES) at low coverage for mutation detection followed by capture-probe sequencing for validation. Our goal was to select the same number of controls, good responders and poor responders, but due to the availability of samples and exclusion criteria, we were not able to identify 20 poor responders, instead 15 poor and 25 good responders entered the analysis. In phase II (**b**), samples that failed WES (not shown, see Supplementary Fig. 1) and samples from additional patients without prior WES were sequenced with the same capture-probe panel as in phase I. To balance the number of patients in the responder groups, preferentially poor responders were added. When samples from phase I and II combined, a total of 86 patients entered the downstream analysis, of which 77 are paired samples (see also Table 2). CONSORT diagram is simplified; a more detailed version can be found in Supplementary Fig. 1. (**c**) Mutation type of all validated mutations in the exome of 59 patients from phase I and (**d**) number of mutations in each patient by responder groups. Identical mutations found in the baseline and surgery sample of the same patients appear once in this figure only.

When phase I was initiated, 148 patients from POETIC (CRUK/07/015) had paired baseline and surgical (2 weeks) RNA*later*-preserved samples available. To focus on a comparison between particularly poor responders and good responders, we excluded treated patients with Ki67 decrease between 60 and 75% (*n*=34, Methods). After quality assessments, we found 60 eligible sample pairs. Our goal was to choose equal numbers of good and poor responders, but in these pairs only 15 poor responders were found. Therefore, all 25 available good responders were included for a set of 40 treated patients. Together with the 20 pairs from the POETIC untreated control group, these constituted the 60 patient cohort of phase I (Fig. 1a; Supplementary Fig. 1). The patient demographics of samples from phase I are described in Supplementary Table 1.

To increase the statistical power to examine common events in AI-treated patients, phase II was subsequently conducted including sample pairs that had become available during continual conduct of the POETIC trial. From 108 available pairs of RNA*later*-preserved samples, we excluded controls (*n*=19) and in keeping with phase I, we excluded samples not falling into either the good or poor responder category (*n*=19). All 18 available poor responding patients were retained even if one sample of the pair did not meet our criteria (12 pairs, 6 singles) together with 10 good responders paired samples selected based on when they were received first in chronological order (Fig. 1b).

The demographics of all 86 patients in this study are described in Table 1.

### Mutation discovery in phase I of the study

Whole-exome sequencing (WES) was performed on tissues at baseline and at surgery and on blood from 60 patients (180 samples in total) for initial mutation discovery. This achieved a median coverage of 38 × (germline 39 × , tumour 37 × ; Supplementary Data 1); 11 tumour samples including both from one patient (P033) were excluded due to low coverage. We identified a total of 6,910 somatic mutations in the remaining tumour samples from 59 patients.

### Mutation validation in phase I of the study

To validate the mutations from WES, we performed targeted re-sequencing at higher depth on all 59 patients (excluding 11 tumour samples and one blood from patient P033, 168 samples in total) from above (Supplementary Fig. 2). Therefore, we designed a capture-probe panel covering all potential somatic mutations discovered from WES. In addition, the entire coding region of 77 previously described breast cancer-related genes were added to the panel (Supplementary Table 2). Seven samples attained low coverage; however, six were sequenced successfully a second time (with samples from phase II, P003 surgery had to be excluded, mean coverage of 7 × ). The remaining 167 samples had median coverage 105 × (germline 110 × , tumour 100 × ; Supplementary Data 1). Of these, 52 were baseline and 56 were surgical samples consisting of 49 pairs: 17 control, 11 poor and 21 good responder pairs (Table 2).

The targeted re-sequencing validated 4,232 somatic mutations across the 59 patients that were classified as tier 1 (variants in the coding regions of annotated exons, canonical splice sites and RNA genes). Without counting identical mutations in paired samples, the number of validated mutations was 6,283 mutations across 108 tumour samples (Supplementary Fig. 3; Supplementary Data 2). These affected 3,388 genes; the majority of mutations were missense (63%) or silent (23%) (Fig. 1c). The mean number of mutations per patient with paired exome sequencing was 79.5 (median 49, interquartile range, 33.0–91.5; Fig. 1d).

Two patients were outliers based on their low mutation count (≤8 mutations in both baseline and surgical samples) in the target area. There were two other pairs of samples with extreme differences in their mutation counts between baseline and surgery: 1 versus 407 (P035, control) and 86 versus 596 (P045, good responder). To exclude sequencing bias, these samples were sequenced a second time to over 200 × median combined coverage per sample. The plot of VAFs between the two runs showed high correlations (*r*=0.85–0.92, Pearson correlation) between the replicates indicating high reproducibility (Supplementary Fig. 4c–f). Despite the higher coverage, many mutations were found in only one or other sample of these pairs (Supplementary Fig. 4a–b), suggesting that these discordances may have been due to normal tissue contamination. This is supported by tumour purity estimation on WES data of these samples (Supplementary Fig. 4).

### Mutational load from phase I samples

**Figure 2:**
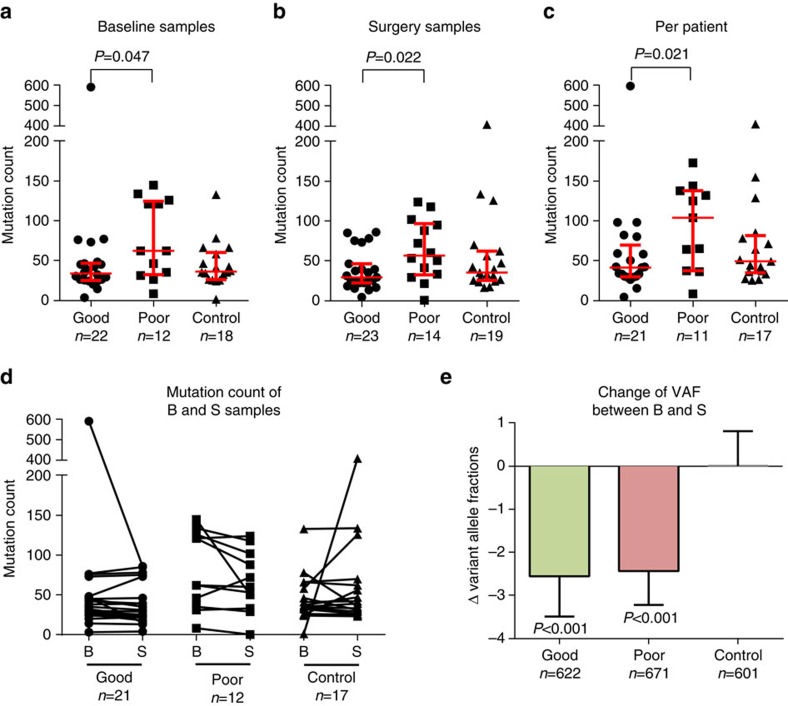
Differences of mutation counts and treatment effects. Analysis on the mutation load of samples with exome-wide mutation profile from phase I. (**a**–**b**) Poor responder showed significantly more mutations than good responder on baseline (B) and surgery (S). (**c**) Also the number of mutations on a per-patient basis (mutations from B and S samples combined, counting identical mutations once only) was significantly higher in poor responders. Median and interquartile ranges are shown as bars. (**d**) No significant difference between the B and S mutation counts within responder groups between each of the 49 paired samples. (**e**) Good and poor responders showed a significant, but low reduction of the mean variant allele fractions (VAFs) of single-nucleotide variants between B and S. Whiskers show 95% confidence interval. Significance was tested by Mann–Whitney test.

For samples in phase I, all potential somatic mutations discovered by WES were added to the capture-panel for validation. This allowed an evaluation of their exome-wide mutational load (that is their total number of mutations). At baseline and at surgery, there was a significant higher mutational load in samples from poor than good responders (median 62.0 versus 33.5, *P*=0.047; Fig. 2a and median 56.5 versus 29.0, *P*=0.022, Fig. 2b; Mann–Whitney test). Controls showed similar mutation numbers to good responders. There was no significant difference between baseline and surgical samples in mutation counts within the good responders, poor responders or control (Fig. 2d). However, considering all 32 treated pairs as a group there was a minor but statistically lower median count after treatment (median baseline 43.5 versus surgery 37.0, median of differences −2, *P*=0.019, Wilcoxon signed-rank test). This significance was retained but weaker after exclusion of the two patients with extreme differences (P035 and P045) from the analysis (*P*=0.034). Given that the treatment-related differences between baseline and surgery were minor, we merged the mutations in each of the pairs of samples and created a count of unique mutations per tumour giving a value for 49 tumours. Similar to the comparisons described above and shown in Fig. 2a,b, we found that poor responders had significantly more mutations than good responders (median 104 versus 41, *P*=0.021; Fig. 2c, Mann–Whitney test).

We compared the VAFs of mutations between the baseline and surgical sample in all tumours and observed correlations up to 0.86 (Pearson correlation, Supplementary Fig. 5). The VAFs of mutations found in both samples of a pair were significantly lower in surgical than baseline samples for good (median baseline 29.2 versus surgery 26.3, *P*<0.001, Wilcoxon signed-rank test) and poor responders (27.0 versus 24.7, *P*<0.001) but not control pairs (27.0 versus 26.5, *P*=0.573; Fig. 2e).

### Mutational clusters from phase I of the study

**Figure 3:**
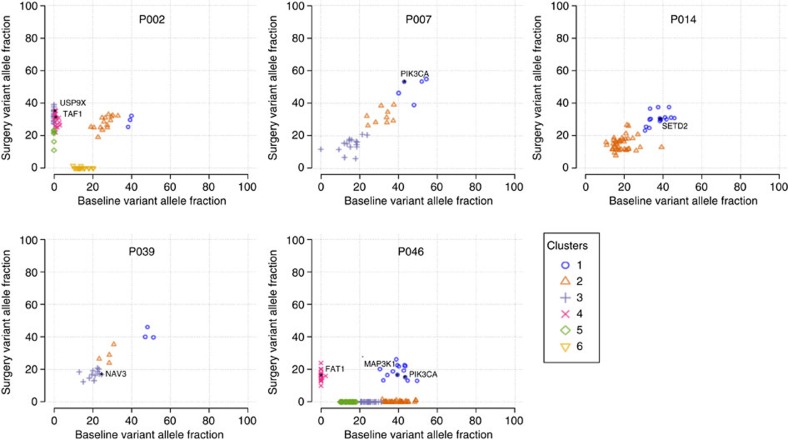
Intra-tumour heterogeneity. Five examples with clear intra-tumour heterogeneity are shown (Supplementary Figs 6–8 for plots of all samples). Some patients had clusters present in both samples (P007, P014 and P039), while others had several clusters that were found in either the baseline or surgery sample (P002 and P046). The variant allele fractions of mutations are shown. Whole-exome sequencing was used for copy-number assessment and only mutations in copy-number neutral regions were plotted. Colours indicate assigned clusters by SciClone (Methods). Cancer-related genes listed in Supplementary Table 4 are labelled in the plots.

We compared the VAFs between baseline and surgical samples to identify mutational clusters which may represent subclones using SciClone^7^ (Methods). SciClone analysis was possible in 40 cases: 11 controls, 20 good and 9 poor responders (Supplementary Figs 6–8). The median number of identified clusters was 3; the maximum number was 6. Five examples are shown in Fig. 3 selected based on a relatively large number of clusters. We did not perform statistical comparisons of the cluster number between the responder groups because of the small sample size. Visual inspection and comparison of SciClone plots did not reveal differences in the degree of heterogeneity between good and poor responders with both groups having patients showing low and high heterogenic sample pairs. In most pairs, the clusters were represented in both samples of the pair (for example, P007, P014 and P039; Fig. 3). In ∼30%, there was clear representation of one or more clusters in only one sample of the pair (for example, P002 and P046; Fig. 3). These exclusive clusters were found in both baseline and surgical samples of all three groups. In these cases, we still found that at least one cluster, usually the one with mutations having the highest VAFs in both samples, which was present in both samples of the pairs.

### Mutation detection in phase II

The capture-probe panel from phase I was used on additional samples from 28 patients (Fig. 1b) and 8 samples from phase I where WES was unsuccessful, but enough DNA was available. Sequencing of one sample from phase I was unsuccessful. In concordance with the analysis in phase I, we excluded germline mutations based on their sequenced matched blood. The median coverage for these samples was 91 × (germline 103 × , tumour 76 × ; Supplementary Data 1). One patient was excluded from further analysis because of a different single-nucleotide polymorphism (SNP) profile (P085; Supplementary Fig. 9). The mutation count for the remaining 27 patients without prior WES discovery was inevitably much lower than for phase I samples (mean 6.4, median 5.0 mutations per patient, interquartile range, 3.0–6.0, Supplementary Fig. 10) as only few mutations in the phase II were found outside the 77 breast cancer-related genes. As for phase I, we only used tier 1 mutations for further analyses (Supplementary Data 3).

### Frequently mutated genes

We combined the mutation data from phase I and II to identify frequently mutated genes in the samples of the 86 patients in our data set (Table 2). Six of the 77 breast cancer-related genes were mutated in 10% or more of the patients. In decreasing frequency, these were *PIK3CA* (37%), *TP53* (26%), *CDH1* (14%), *MLL3* (14%), *ABCA13* (12%) and *FLG* (10%). The top three genes are also the most frequently mutated genes in ER+, post-menopausal breast cancers in TCGA^8^ (Supplementary Table 3). The frequency of mutations in *PIK3CA* and *CDH1* was very similar to the TCGA cohort, but the other four genes showed higher frequency in our data set, especially *ABCA13* with 12% compared with 4% in TCGA. We assessed whether good or poor responders were significantly associated with mutations in *ABCA13* or other frequently mutated genes, but we did not find such an association (6/27 versus 2/31, *P*=0.258, Fisher's exact test, not shown for other genes). Apart from the top three frequently mutated genes (*PIK3CA*, *TP53* and *CDH1*), only *GATA3*, *RYR2* and *MAP3K1* are mutated in >5% of patients in TCGA (9%, 6% and 9% of tumours, respectively). For these, we found similar frequencies in our set (7%, 6% and 5%, respectively). The most recurrent amino-acid changes in our patients were H1047R (in 14 patients) followed by E545K (5 patients) in *PIK3CA*. For the majority of the frequently mutated genes, missense was the most common amino-acid change. Exceptions were *CDH1* with predominantly frameshift mutations (12 frameshift, 1 missense and 1 nonsense) and *MLL3* with nonsense mutations (14 nonsense, 4 missense and 1 frameshift).

**Figure 4:**
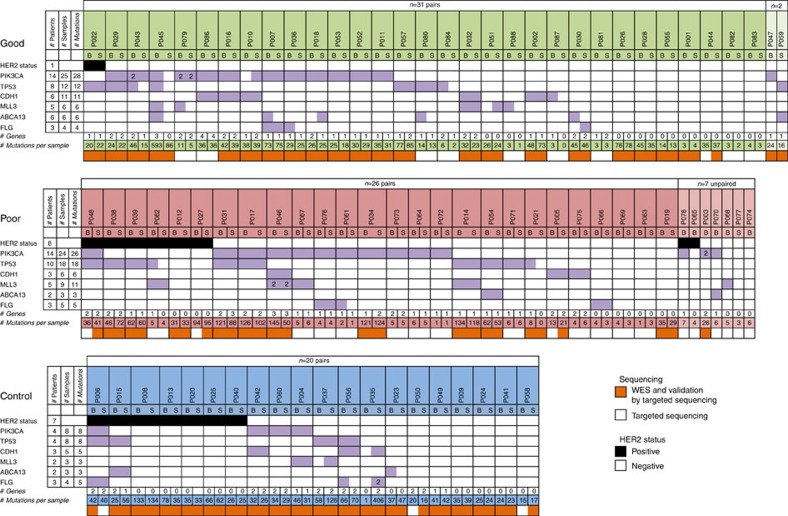
Frequently mutated genes. Sample matrix for genes with mutations in 10% or more of the patients. All 163 tumour samples from 86 patients (including 77 pairs) with baseline (B) and surgery (S) sample are shown. For phase I samples, the bottom row shows if the sample successfully underwent whole-exome sequencing and therefore mutations identified in this sample were added to the capture-probe panel. The *TP53* mutation of P038 and one of each mutation of *MLL3* for P046 were not identical between B and S. The overall concordance between B and S samples of patients was 71%.

There was at least one mutation in a frequently mutated gene in 53 of the 77 pairs (Fig. 4). In all but 22 cases, the mutations in frequently mutated genes were identical for both samples of the pair giving a 54% concordance. In these pairs, 28 sites were identified as discordant, although 14 of these showed a measurable frequency (but not reaching statistical significance) in the other sample of the pair. The other discordant sites showed no frequency in the other samples of the pair, but all had a coverage >50 × . The mutation status per patient (identical mutations or wild type (WT)) of the 6 frequently mutated genes was concordant in 71% of the complete set of 77 pairs. For individual genes, the concordance was higher for *PIK3CA* (3/27 discordant/concordant, 90%) and *TP53* (7/15, 68%) compared with the less frequently mutated genes *ABCA13* (6/2, 25%) and *FLG* (6/4, 40%). Also, the VAF of mutations in *PIK3CA* (median baseline/surgery 30.3/28.8%) and *TP53* (33.3/33.1%) were generally higher than for *ABCA13* (15.5/11.1%) and *FLG* (12.3/13.5%), which were lower than the overall median of 25.7%.

Mutations in breast cancer-driver genes listed by DriverDB^9^ were found in 65 of the 77 sample pairs with a median of two driver gene mutations per sample (Supplementary Table 4). In 25 pairs all driver mutations were identified in both samples. Twelve pairs had none of their driver mutations shared, resulting in an overall concordance of 54%.

### TP53 and HER2

**Figure 5:**
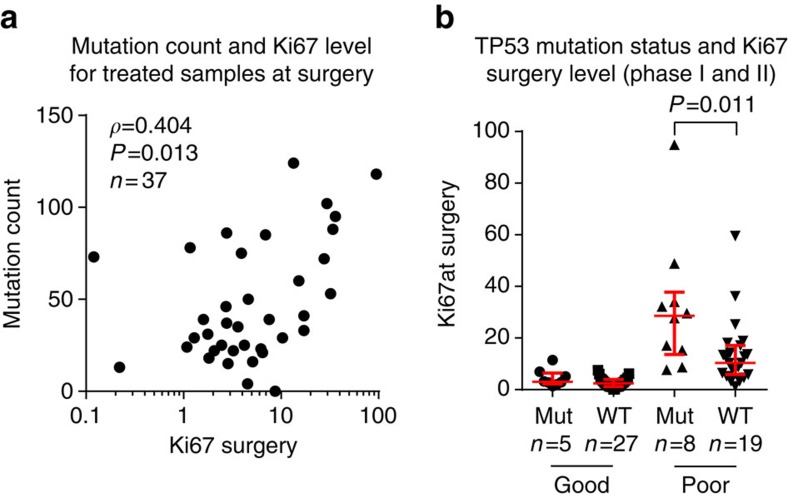
Relation of mutations to Ki67. (**a**) Correlation of mutations counts to the Ki67 level was highest for treated samples at surgery. (**b**) On the combined set from phase I and phase II, the Ki67 level of poor responders was significantly higher for patients with mutated *TP53* (mut) than wild-type *TP53* (WT). This was not seen for good responders, although Ki67 level for *TP53*-mutated patients was higher on baseline (Supplementary Fig. 11). Significance tested by Mann–Whitney test, red lines show median and interquartile ranges.

Non-functional *TP53* can lead to DNA damage accumulation^10^. Therefore, we compared the mutational load of samples from phase I by their *TP53* mutation status and found a significantly higher load for mutated samples (median WT 37 versus mutant 64.5, *P*=0.017, Mann–Whitney test). For the samples from phase I, the mutational load correlated weakly with Ki67 level at baseline (*r*=0.31, *P*=0.02 Spearman correlation), but a moderate correlation was found for the treated samples at surgery (*r*=0.40, *P*=0.01; Fig. 5a). Poor responders and *TP53* are both associated with higher mutational load: using the combined set of patients (phase I and II), we hypothesized that poor responders were more likely to have a *TP53* mutation compared with good responders, but this hypothesis was rejected (10/23 versus 8/25, *P*=0.8, Fisher's exact test). However, we found a significantly higher Ki67 baseline level for *TP53*-mutated samples (Supplementary Fig. 11) for both good (median WT 16.9 versus mutated 36.7, *P*=0.020, Mann–Whitney test) and poor responders (median WT 15.9 versus mutated 32.3, *P*=0.006). This difference was lost after treatment for the good, but persisted for poor responders (median WT 10.3 versus mutated 28.7, *P*=0.011, Fig. 5b).

In HER2+ and HER2− tumours, the median mutational load was 64 and 42, respectively (*P*=0.180, Mann–Whitney test). There was a higher than expected HER2+ rate among the control samples (35% in this data set, expected rate in an ER+ population is ∼10% (ref. 11)).

A significant decrease in the cellularity between baseline and surgery samples was found for good, but not poor responders or controls (Supplementary Fig. 12) as reflected by the total number of cells per high-powered field in the Ki67 analysis. The type of biopsy taken at surgery (core-cut or resection) did not differ statistically between any responder groups and did not explain differences in cellularity for good and poor responders (Supplementary Fig. 13).

## Discussion

Our primary goal was to identify DNA changes that relate significantly to the response of ER+ breast cancer to short-term oestrogen deprivation using AIs. Although the pre-surgical setting was ideal for this purpose, little is known about the true, as opposed to theoretical, impact of tissue heterogeneity on mutational profiling from the small tumour biopsies that are available for mutation profiling studies of clinical material. Our data on reproducibility are critical for a valid understanding of the current study and the many other studies of this type.

Very few data have been published on the genomic heterogeneity of multiple cores taken from the same breast tumour. The correlations of VAFs from two samples from five breast tumours reported by Ellis *et al*.^6^ (*r*=0.74–0.94) were consistent with the majority of comparisons in the current analysis but in our larger set the correlations were much lower for some of our cases (Supplementary Fig. 5). Preliminary data were recently reported on 13 patients with multiple (7–17) spatially separated samples of primary breast cancer (ER+ and other types)—heterogeneity was observed within the samples even of cancer-driver mutations^12^. Yates *et al*.^13^ reported heterogeneity in 8 out of 12 treatment-naive breast cancers based on eight spatially separated biopsies from each tumour.

Most pairs in our study showed several clusters (potential subclones) present in both samples, but in ∼30% of the cases, we also found sample pairs with several clusters being exclusive to either sample and therefore spatially separated in the same tumour. However, these pairs shared at least one cluster, usually the one with the highest VAFs, indicative of a common founding clone with driving cancer mutations^14^. Although clusters exclusive to one sample were often present in a small proportion of sequenced cells, each subclone potentially has different adaptive properties and might become the dominant clone due to selection from treatment^15^,^16^. Clusters disappearing or becoming more prominent in the treatment group could be indicative of such a selection. In our data, it is unlikely that the exclusive clusters occur due to the selection from AI treatment, since we found exclusive clusters in the control group as well and AI treatment had a very modest effect on cellularity in the 2 weeks of this study.

Reduced heterogeneity was found after 6 months of AI treatment^17^. In our data, after much shorter time, we found that the number of mutations and the VAFs were slightly but statistically significantly lower in the surgical samples of treated compared with control patients, possibly indicating a modest treatment effect. Such a small effect was consistent with the slow rate of clinical response of tumours to endocrine therapy. This is dependent on cytostasis and not on enhanced cell death such that tumour shrinkage is rarely apparent over a 2-week time period. In the good responder group, we noted that a minor loss of cellularity occurred over the 2-week period based on field counts of nuclei. Reduced cellularity could conceivably make the microdissection we carried out for all tissue sections before genomic analysis more difficult and thereby lead to greater non-malignant cell contamination potentially reducing the sensitivity to detect variants. These results are therefore consistent with the slightly decreased number of mutations in the surgical samples being at least in part an artefact of the lower malignant cell purity in the dissected material from the surgical samples. Given that the median loss between baseline and surgical samples from AI-treated patients was only two mutations, we rationalized that surgical samples even from these were sufficiently unaffected by treatment to be acceptable as representative of the untreated state. Merging mutation data from baseline with surgical samples including those from treated patients should provide more comprehensive information on the mutational landscape of a tumour than single cores.

Modest coverage for WES might have missed mutations with low VAF, especially mutations present at very low frequency in both samples of a pair. These mutations therefore could not be integrated in the panel and subsequently are missing in the final set of mutations and subclones. To maximize the number of mutations in the capture-panel, we used the union of several variant callers on the WES data to detect potential somatic mutations. Further, we included the entire coding sequencing of 77 breast cancer-related genes in the panel to be able to detect mutations in these independent of the discovery step. We used the same capture-probe panel for additional samples in phase II of this study. Unlike phase I, the panel was not specifically designed to validate mutations found in the discovery stage. Therefore, in phase II, far fewer mutations per sample were found outside the 77 breast cancer-related genes, emphasizing the individuality of the mutational profile of each breast cancer tumour^18^. For the combined set of samples from phase I and II, we therefore exclusively concentrated on the 77 breast cancer genes present on the targeted-panel and did not perform analyses based on mutation count or subclonality with these.

As expected, the most frequently mutated genes across the 86 patients were the breast cancer-driver genes *PIK3CA* and *TP53* (ref. 6)*. CDH1* (ref. 19) and *MLL3* (ref. 20) are also frequently mutated genes known to be linked to breast cancer. The genes *FLG* and *ABCA13* are less studied, but *FLG* was shown to be amplified in a subset of breast cancers^21^. The frequency of patients with mutations in *ABCA13* was about threefold higher compared with post-menopausal ER+ breast cancer tumours from TCGA^8^. A reason for this could be the selection of patients based on good and poor response; however, we did not find significant differences between good and poor responders in terms of the mutation frequency of *ABCA13* or other frequently mutated genes. *GATA3* was not in the top list of frequently mutated genes, but its frequency was similar to that in TCGA. It was suggested that *GATA3* mutations might be a positive prediction marker for AI response based on Ki67 decline^6^. Our data cannot support this finding, but the statistical power with six mutated patients is low.

We saw low correlations for some samples based on the VAF values of all mutations in a sample pair. However, the mutation status of frequently mutated genes in the present data was found to be consistent within pairs in 76% of cases. Thus, in a majority of cases, the profile of mutations in the genes would be represented by one core. However, in about one in four patients this would not be the case and a single core-cut would have missed a potentially important gene mutation. We noted higher discordance and lower VAFs for mutations in less frequently mutated genes (*ABCA13* and *FLG*). This suggests that these mutations are subclonal, but might have important functions upon selective pressure. However, mutations at lower VAF are also more difficult to detect, which might in part explain the lower concordance for these mutations. We also analysed the concordance for the more numerous driver genes listed in DriverDB and we found a lower concordance of 54% between all pairs.

To study the impact of mutational profile on response to AI treatment, patients at the extreme ends of the Ki67 response spectrum were chosen as poor or good responders from the available patient sample set. Change in Ki67 after 2 weeks is a validated end point for benefit from adjuvant endocrine therapy, while the value of Ki67 after 2 weeks is prognostic for recurrence-free survival^22^. Ellis *et al*.^6^ related the mutational profile to resistance to AI in 77 patients using Ki67, defining resistance as on treatment Ki67>10% irrespective of starting level. According to this definition, four patients in our data set would have been categorized as good responders despite exhibiting a minimal Ki67 decrease. Nonetheless, there is generally good concordance between these two definitions and the major conclusions on AI resistance from the current study and the Ellis study are similar.

We excluded 7% of patients who were categorized as poor responders according to Ki67 decrease due to a lack of E2 suppression. It is not known whether this was due to poor compliance or poor pharmacologic response, but whichever is the case this highlights the importance of measuring primary pharmacological response to avoid intensive molecular investigation of tumours for mechanisms of resistance when the expected pharmacological perturbation is absent.

The relatively low frequency of mutations in most genes in primary breast cancer means that large studies are required to define reliable associations with response/resistance to therapy even in pre-surgical studies such as POETIC where biological response is measurable in all treated patients (in contrast to adjuvant therapy). Nonetheless, we found a reduced suppression of Ki67 for *TP53*-mutated tumours within the poor responder group, which supports the finding by Ellis *et al*.^6^ who reported a greater suppression of Ki67 by letrozole in WT than *TP53*-mutated tumours. This indicates at least in part that *TP53* mutations are a marker for poor response to AI in addition to being a marker for poorer outcome for ER+ breast cancer. We also found a significant association of mutated *TP53* with increased mutational load. For *TP53*, this is consistent with it being an important DNA repair gene, malfunction of which may lead to general genomic instability and an increase in mutations. The association of these factors with high mutational load was recently reported by Haricharan *et al*.^23^

It could be expected that poor responders to endocrine therapy might exhibit greater genomic heterogeneity given its potential to provide multiple pathways of resistance, a hypothesis supported by the larger number of mutations found in poor responders in this study. The clear presence of subclonality and multiple driver mutations in some of these early breast tumours does indicate the potential for some subclones to be selected preferentially during hormonal treatment and to drive the clinical regrowth of a partially responsive tumour. Identification of such subclones or mutations requires further studies on a later time point when the effect of treatment would be greater than that at 2 weeks.

In conclusion, this study demonstrates that multiple subclones are present even in early ER+ breast cancer. In most cases, the subclones and their constituent mutations are represented in different core-cuts from the same tumour but in ∼30% of the tumours mutations are exclusive to one of the core-cuts. Increased mutational load is associated with poorer antiproliferative response to AI possibly driven by mutations in *TP53*.

## Methods

### Patients and tissues

The design and goals of the POETIC trial (CRUK/07/015) have been published^2^. In brief, post-menopausal patients with primary ER and/or PgR-positive (according to local testing) breast cancer in over 120 centres across the United Kingdom were randomized 2:1 to receive or not receive an AI (anastrozole 1 mg per day or letrozole 2.5 mg per day) for a 4-week period starting 2 weeks before surgery.

Core-cut biopsies (14 G) and either core-cuts or part of the excision sample were collected at baseline and surgery, respectively, and fixed in formalin. Additional core-cuts were collected into RNA*later* (Qiagen) at both time points. Whole blood was collected for germline DNA analysis, baseline and surgical plasma for estradiol analysis.

The trial was approved by the NRES Committee London—South East. All patients gave informed consent for DNA sequencing.

### Biomarker analyses

Ki67% staining (MIB-1 clone code n. M7240, DAKO UK Ltd; working dilution 1:40) was the primary biomarker end point for the POETIC trial and was centrally analysed on all formalin-fixed samples using a single protocol (either core-cut in formalin-fixed, paraffin-embedded or excision specimens in formalin-fixed, paraffin-embedded) as previously described^22^. All staining was performed on a Dako autostainer using strict adherence to a single staining protocol. Haematoxylin and eosin staining was used to exclude samples with low tumour purity (<40%).

HER2 status was measured locally using immunohistochemistry and/or *in situ* hybridization^24^. Biomarker results are shown in Supplementary Table 5.

ER expression of baseline specimens was measured by immunohistochemistry (6F11 clone code n. NCL-L-ER-6F11, Leica Biosystems Ltd; working dilution 1:50) on formalin-fixed samples^25^. Patients were excluded from this substudy if they were described as ER negative (<1% positive staining of tumour nuclei).

Cellularity was measured by 10 × 10 mm eye-piece graticule with × 40 objective graticule. Nuclei were counted within the grid of at least five fields and the mean values from these measurements were used.

Patients with unsuppressed estradiol upon treatment were excluded.

### Sample selection

In phase I, samples were selected with the aim of having equal numbers of control patients, definite poor responders defined as having a Ki67 decrease of <60% between baseline and surgery and good responders with >75% Ki67 decrease. The definition of good responders was selected as being above the mean Ki67 reduction to anastrozole after 2 weeks^26^. Patients with Ki67 decrease between 60 and 75% were excluded to create an efficient design that focused on the extremes of the range of Ki67 responses. Treated patients not showing suppressed post-menopausal levels of plasma estradiol and those with central ER <1% were excluded. For phase II, only treated samples were selected.

### DNA extraction

DNA was extracted from RNA*later*-preserved diagnostic (baseline) and surgical (surgery) 14-G core-cut samples and peripheral blood.

At least eight unstained 8-μm sections were taken from core-cuts embedded in OCT (Cryo-M-Bed, Bright Instruments, UK). Sections were stained with Nuclear Fast Red (0.1% (w/v)) and when necessary needle microdissection was used to achieve >60% pure tumour cells using an adjacent haematoxylin- and eosin-stained section as a guide. DNA was extracted from the sections using the DNeasy Tissue and Blood kit (Qiagen) and from blood using the EZ1 system (LifeTechnologies).

### Exome sequencing for discovery

Cavitation (adaptive focused acoustics, Covaris) was used to fragment the samples. The automated libraries were generated with in-house Illumina kits at Washington University, MO, with reagents supplied by NEB and indexed via PCR. LucigenDNATerminator kit (end repair), NEB Klenow (adenylation), NEB Quick Ligase (ligation, Illumina's Multiplexing Adapters) and NEB Phusion (PCR enrichment, libraries were indexed via PCR (PCR1.0, PCR2.0 and index primers), AMPure beads were used for enzymatic purification and size selection). Manual libraries were generated with KAPA Library Preparation with standard PCR library amplification (KK8201) and libraries were indexed during ligation with TruSeq LT adaptors. LabChip GX was used for library quantitation as well as quality control. Size selection was conducted using AMPure beads. Ten libraries were pooled pre-capture. Each library pool was captured using NimblegenSeqCap EZ Human Exome Library v3 (with requisite SeqCap EZ hybridization and wash kits) and sequenced on two lanes of the IlluminaHiSeq 2000 with v3 chemistry (2 × 100bp).

Sequence data were aligned to reference sequence build GRCh37-lite-build37 using bwa version 0.5.9 (ref. 27; params: -t 4 -q 5) then merged using picard version 1.46 (http://picard.sourceforge.net), then deduplicated using picard version 1.46.

Single-nucleotide variants were detected using the union of three callers: (1) samtools version r963 (ref. 28; params: -A -B) intersected with Somatic Sniper version 1.0.2 (ref. 29; params: -F vcf -q 1 -Q 15) and processed through false-positive filter v1 (params: --bam-readcount-version 0.4 --bam-readcount-min-base-quality 15 --min-mapping-quality 40 --min-somatic-score 40), (2) VarScan version 2.2.6 (ref. 30) filtered by varscan-high-confidence filter version v1 and processed through false-positive filter v1 (params: --bam-readcount-version 0.4 --bam-readcount-min-base-quality 15 --min-mapping-quality 40 --min-somatic-score 40) and (3) Strelka version 0.4.6.2 (ref. 31) (params: isSkipDepthFilters=1).

InDels were detected using the union of four callers: (1) GATK somatic-indel version 5,336 (ref. 32) filtered by false-indel version v1 (params: --bam-readcount-version 0.4 --bam-readcount-min-base-quality 15), (2) pindel version 0.5 (ref. 33) filtered with pindel false-positive and vaf filters (params: --variant-freq-cutoff=0.2), (3) VarScan version 2.2.6 (ref. 30) filtered by varscan-high-confidence-indel version v1 then false-indel version v1 (params: --bam-readcount-version 0.4 --bam-readcount-min-base-quality 15), and (4) Strelka version 0.4.6.2 (ref. 31; params: isSkipDepthFilters=1).

### Targeted sequencing for validation

All of the variants (*n*=6,910) identified in the discovery set excluding those in low-coverage samples were chosen for validation at greater depth as well as exons of a set of 77 breast cancer-related genes of interest (Supplementary Table 2). Probes were designed to target the variants within 13,372 regions of 6,737 genes covering a total of 2,645,703 bp.

Reads were aligned as described above for exome sequencing. Single-nucleotide variants were detected using VarScan version 2.2.6 (with parameters --min-var-freq 0.08 --*P*-value 0.10 --somatic-*P*-value 0.01 --validation) and filtered by Varscan-high-confidence version v1, then false-positive version v1 (with parameters: --bam-readcount-version 0.4 --bam-readcount-min-base-quality 15).

InDels were detected using the union of three callers: GATK somatic-indel version 5,336, pindel version 0.5 (filtered by pindel-somatic-calls version v1, then pindel-read-support version v1) and VarScan version 2.2.6 (filtered by varscan-high-confidence-indel version v1, then false-indel version, with parameters: --bam-readcount-version 0.4 --bam-readcount-min-base-quality 15).

In addition to using matched normal for germline detection, sites that were present in at least 0.1% of the general population according to the 1000 Genomes Project^34^ or NHLBI GO Exome Sequencing Project were removed from further analysis.

All somatic events from re-sequencing were manually reviewed using IGV^35^.

### SNP profile

Samples were confirmed as being derived from the same patient by correlation of SNPs (Supplementary Fig. 9) based on the sequencing data. Therefore, all samples have been profiled based on over 500 SNPs from dbSNP version 138 within the area of the capture-panel. The genotypes at genomic position were derived using samtools^28^. SNPRelate^36^ was used to cluster the samples and generate the dendrogram using default parameters.

### Subclonal analysis

Clonal architecture was inferred using SciClone version 1.0.4 (ref. 7; params: minimumDepth=50) using copy number and loss of heterozygosity (LOH) calls derived from Varscan (params: loh-cutoff=0.95, min-loh-probes=10, min-mapping-quality=10, min-coverage=20, min-segment-size=25, max-segment-size=100, undo s.d.=4). Samples with low mutation count failed clustering and were excluded from the analysis. SciClone plots were annotated with frequently mutated genes from Supplementary Table 6.

### Comparison with known driver genes

Validated mutations in the baseline and surgery samples were compared with known driver genes in the DriverDB^9^ database. Therefore, ‘breast' tissue was selected as cancer type and all genes identified by at least two tools were downloaded from the website.

### Estimating tumour purity based on WES

For estimating cellularity based on whole-exome sequencing data Sequenza v2.1.1 (ref. 37) was used. The algorithm was applied for each tumour sample and its matched blood sample. In brief, it first detects germline mutations in the normal sample and then calculates the VAF at the same position in the tumour sample. In the second step, the tumour versus normal depth ratio is calculated with GC content normalization and allele-specific segmentation is performed. Based on a probabilistic model applied to the segmented data, Sequenza calculates possible solutions for cellularity and ploidy of the tumour. The default settings were used for all steps, the cellularity with the highest probability was reported.

### Statistical analysis

Unpaired and nonparametric Mann–Whitney test was used to test the differences of mutation counts between groups. The Wilcoxon signed-rank test was used to test for differences in the mutation counts of paired samples between baseline and surgery and to compare the VAFs between baseline and surgery samples. The associations of *TP53* mutation status and HER2 status between the groups were analysed using Fisher's exact test. Reported *P* values are two-sided and unadjusted; *P* value<0.05 is considered to be significant in this study. The statistical analyses were conducted in GraphPad Prism 6 (Graphpad Software Inc.) and R^38^.

### Data availability

The sequencing data that support this study have been deposited in the European Genome-phenome Archive (EGA) database under accession code EGAS00001001940. The remaining data are available in the article or its Supplementary Files or available from the authors on request.

## Additional information

**How to cite this article:** Gellert, P. *et al*. Impact of mutational profiles on response of primary oestrogen receptor-positive breast cancers to oestrogen deprivation. *Nat. Commun.* **7,** 13294 doi: 10.1038/ncomms13294 (2016).

**Publisher's note:** Springer Nature remains neutral with regard to jurisdictional claims in published maps and institutional affiliations.

## Supplementary Material

Supplementary Information Supplementary Figures 1-13, Supplementary Tables 1-6 and Supplementary References

Supplementary Data 1 Sequencing coverage of each sample. Listed are coverage statistics for each sample for whole exome sequencing and targeted sequencing calculated by GATK's “DepthOfCoverage” using default settings. For each sequencing the total, mean, third quartile, median and first quartile coverage are shown. Additionally, the % of bases with a coverage > 15 is shown. Cells marked in red indicate low coverage samples and therefore were not used for further analysis. Samples with low coverage for whole exome sequencing were not used to design our custom capture-probe panel and were therefore treated as samples from phase II. Samples with low coverage for targeted sequencing were excluded completely from any analysis.

Supplementary Data 2 List of identified mutations and their variant allele fractions from samples of phase I. The “source” column indicates if mutation was called by the somatic variant caller in the baseline, surgery or both samples followed by the variant allele fractions (VAFs) in the samples.

Supplementary Data 3 List of identified mutations and their variant allele fractions from samples of phase II. The “source” column indicates if mutation was called by the somatic variant caller in the baseline, surgery or both samples followed by the variant allele fractions (VAFs) in the samples.

## Figures and Tables

**Table 1:** Clinical data summary of all 86 patients in this study.

	**Response group**					
	**Poor (*****n*****=33)**		**Good (*****n*****=33)**		**Control (*****n*****=20)**	
	***n***	**%**	***n***	**%**	***n***	**%**
*PgR status*						
Positive	20	60.6	26	78.8	16	80.0
Negative	7	21.2	5	15.2	3	15.0
Not known	6	18.2	2	6.1	1	5.0
						
*Histological subtype*						
Ductal	27	81.8	24	72.7	17	85.0
Lobular	3	9.1	5	15.2	2	10.0
Mucinous	1	3.0	1	3.0	0	0.0
Mixed ductal and lobular	2	6.1	1	3.0	1	5.0
Not known	0	0.0	2	6.1	0	0.0
						
*Pretreatment tumour grade*						
G1	2	6.1	0	0.0	3	15.0
G2	14	42.4	22	66.7	9	45.0
G3	10	30.3	4	12.1	5	25.0
Not known	7	21.2	7	21.2	3	15.0
						
*No. of involved lymph nodes*						
N0	20	60.6	20	60.6	11	55.0
N1-3	7	21.2	11	33.3	6	30.0
N4+	6	18.2	2	6.1	3	15.0
						
HER2 status						
Negative	25	75.8	32	97.0	13	65.0
Positive	8	24.2	1	3.0	7	35.0
						
*Pretreatment tumour size (cm)*						
<2	12	36.4	11	33.3	7	35.0
2–5	19	57.6	22	66.7	12	60.0
>5	2	6.1	0	0.0	1	5.0
						
*Surgery tumour size (cm)*						
<2	12	36.4	13	39.4	8	40.0
2–5	20	60.6	20	60.6	10	50.0
>5	1	3.0	0	0.0	2	10.0
						
	**Median**	**IQR**	**Median**	**IQR**	**Median**	**IQR**
Age at randomization (years)	70	61–78	74	62–82	70	59–76
Time from randomization to surgery (days)	19	15–23	17	15–19	18.5	14–23.5

Patient's demographics are separated by poor responder, good responder and control. All analysis based on somatic mutations within 77 breast cancer-related genes are conducted on this set of patients combined from phase I and II (no analysis was conducted on phase II samples only). Analyses on the exome-wide mutational load were performed on samples from phase I with whole-exome sequencing. The demographics of these patients only are shown in Supplementary Table 1.

**Table 2:** Summary of available samples in this study.

**Summary**	**Total**	**Good**	**Poor**	**Control**
*Phase I*				
Samples with mutations discovered by exome sequencing	108	45	26	36
Patients with paired exome sequencing	49	21	11	17
Patients with exome sequencing from either sample	59	24	15	20
Patients with exome sequencing in baseline	52	22	12	18
Patients with exome sequencing in surgery	56	23	14	19
				
*Phase I and II*				
Samples with targeted sequencing	163	64	59	40
Patients with targeted sequencing from either sample	86	33	33	20
Patients with paired targeted sequencing	77	31	26	20
Patients with targeted sequencing in baseline	84	32	32	20
Patients with targeted sequencing in surgery	79	32	27	20

Analyses on the mutational load and the mutational clusters were performed on phase I samples with exome sequencing available. Analyses based on 77 breast cancer-related genes were performed on the combined set of phase I and II samples. As indicated in the text, some analyses were performed on patients with paired baseline and surgery samples only.
