# Supplementary material for: Impact of mutational profiles on response of primary oestrogen receptor-positive breast cancers to oestrogen deprivation
Source: Nat Commun. 2016 Nov 9;7:13294. doi: 10.1038/ncomms13294 (PMC5105193; doi:10.1038/ncomms13294)
Supplement: Supplementary Information — Supplementary Figures 1-13, Supplementary Tables 1-6 and Supplementary References [file ncomms13294-s1.pdf]

Supplementary Figures

Supplementary Fig. 1: CONSORT diagram.

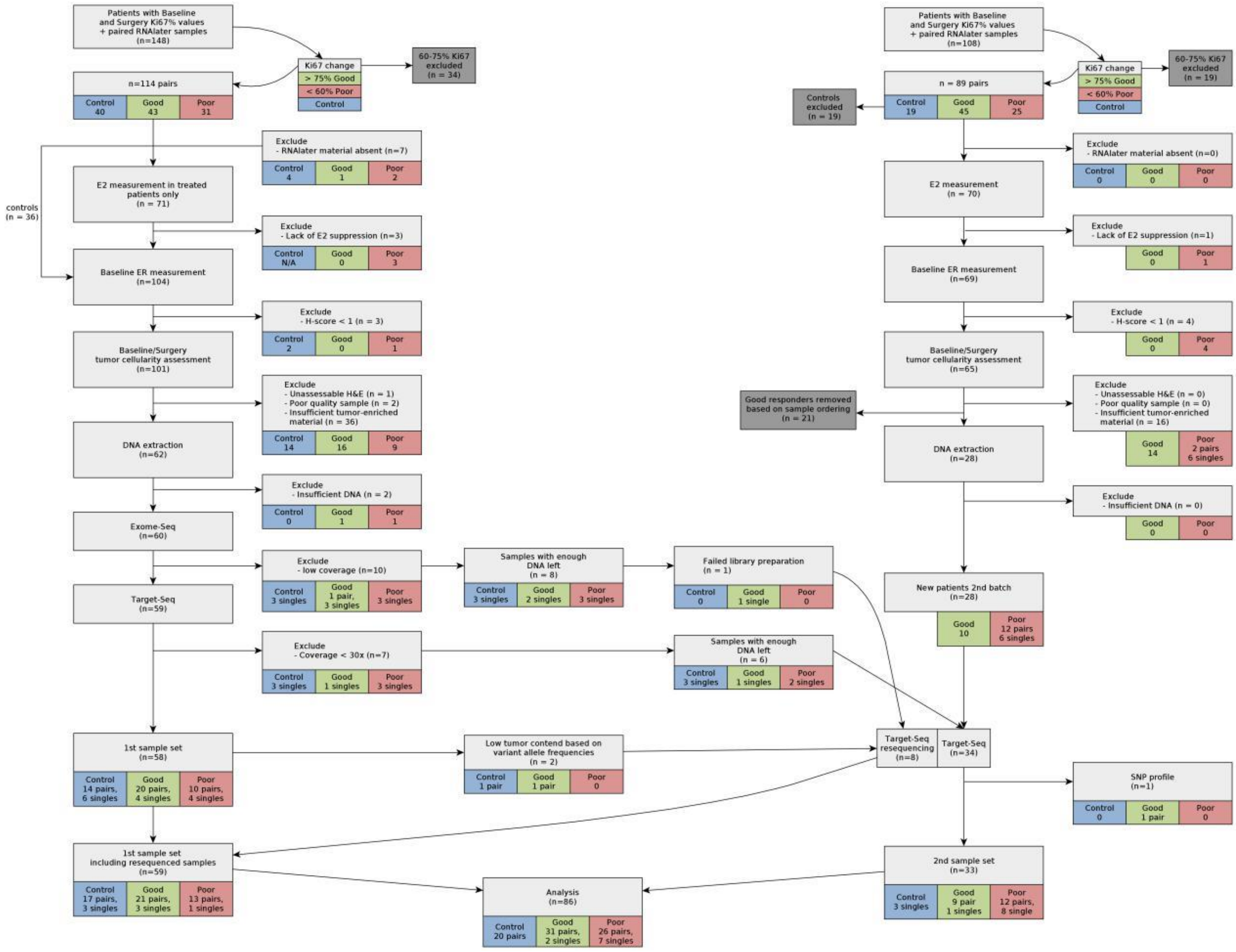

Grey boxes indicate different measurement or analysis steps and criteria for sample exclusions with the number of patients (single or paired). Samples with successful whole exome sequencing, but low coverage during the first capture-probe sequencing (left) were sequenced a second time (“Target-Seq resequencing”) and included in the set of samples from Phase I. Samples that failed whole exome sequencing were sequenced together with the additional samples (right) from Phase II. In both cases the same DNA extract was used, if the amount of left DNA allowed this. Two pairs (1 good responder, 1 Control) were sequenced in 1st set and 2nd set, although coverage was not low to measure reproducibility

of the sequencing (Supplementary Fig. 4). These two pairs therefore appear once in the final analysis only. In total 86 patients entered the downstream analysis, of which 77 are paired samples. For the baseline, a total of 84 samples was available, and for the surgery 79.

Abbreviations: E2, 17β-estradiol; ER, oestrogen receptor; H&E, hematoxylin and eosin.

Supplementary Fig. 2: Sequencing overview.

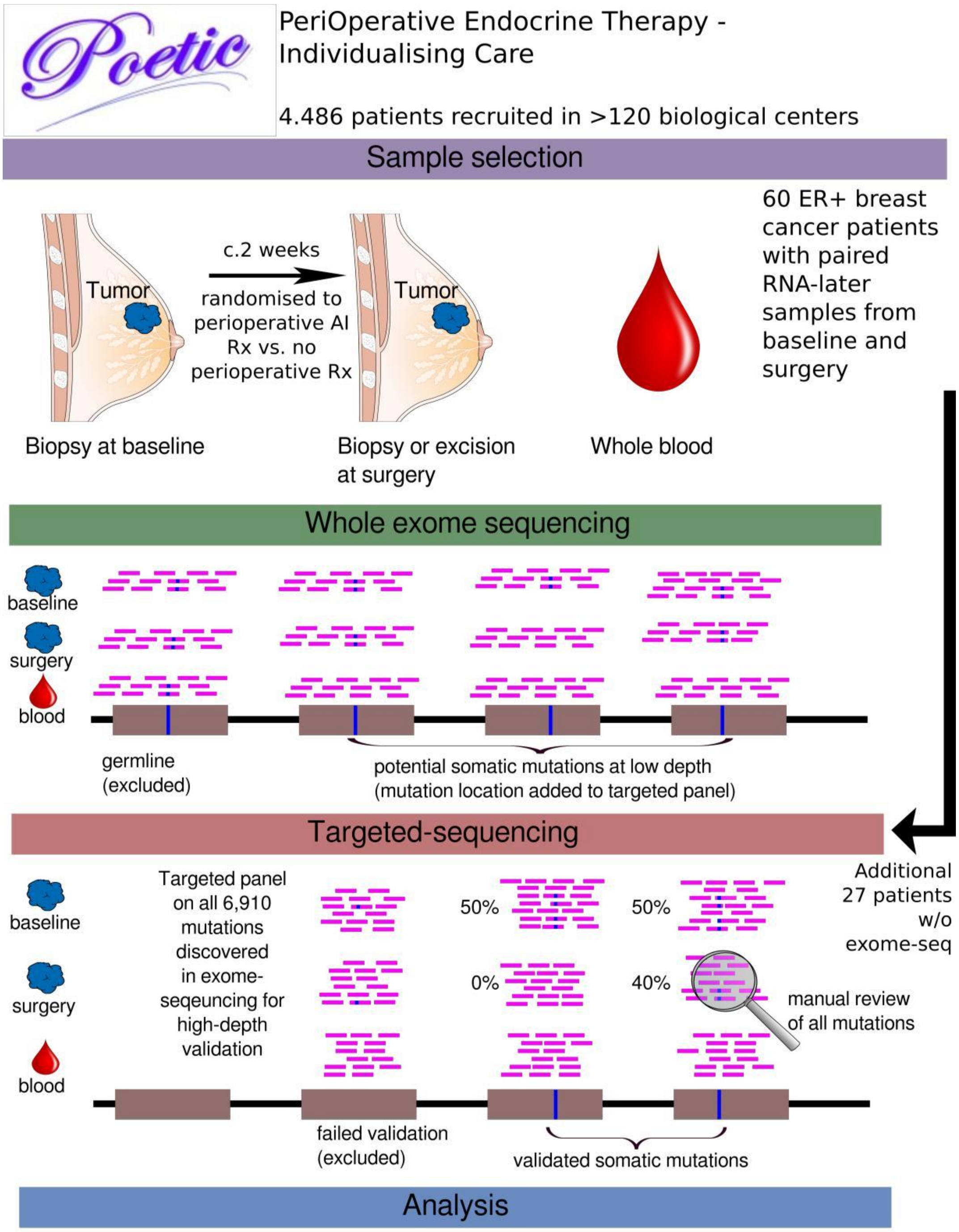

Overview of the sequencing process conducted in this study. Samples were selected from POETIC as described in the main text. Initially 60 patients with paired RNA*later* preserved samples were selected for whole exome sequencing (WES). For each patient the baseline, surgery and blood samples were sequenced at low depth to discover somatic mutations. Germline mutations were excluded based on the blood samples. A capture panel was

designed on all potential somatic mutations found within these samples and additionally 77 breast cancer related genes were added. Samples with low coverage from WES were not used for designing the capture-panel. All samples that underwent exome sequencing plus additional samples from 28 patients were sequenced at high depth for confident somatic mutation calling. Again the matched blood samples from each patient were used to exclude germline mutations.

Supplementary Fig. 3: Mutations per sample and patient.

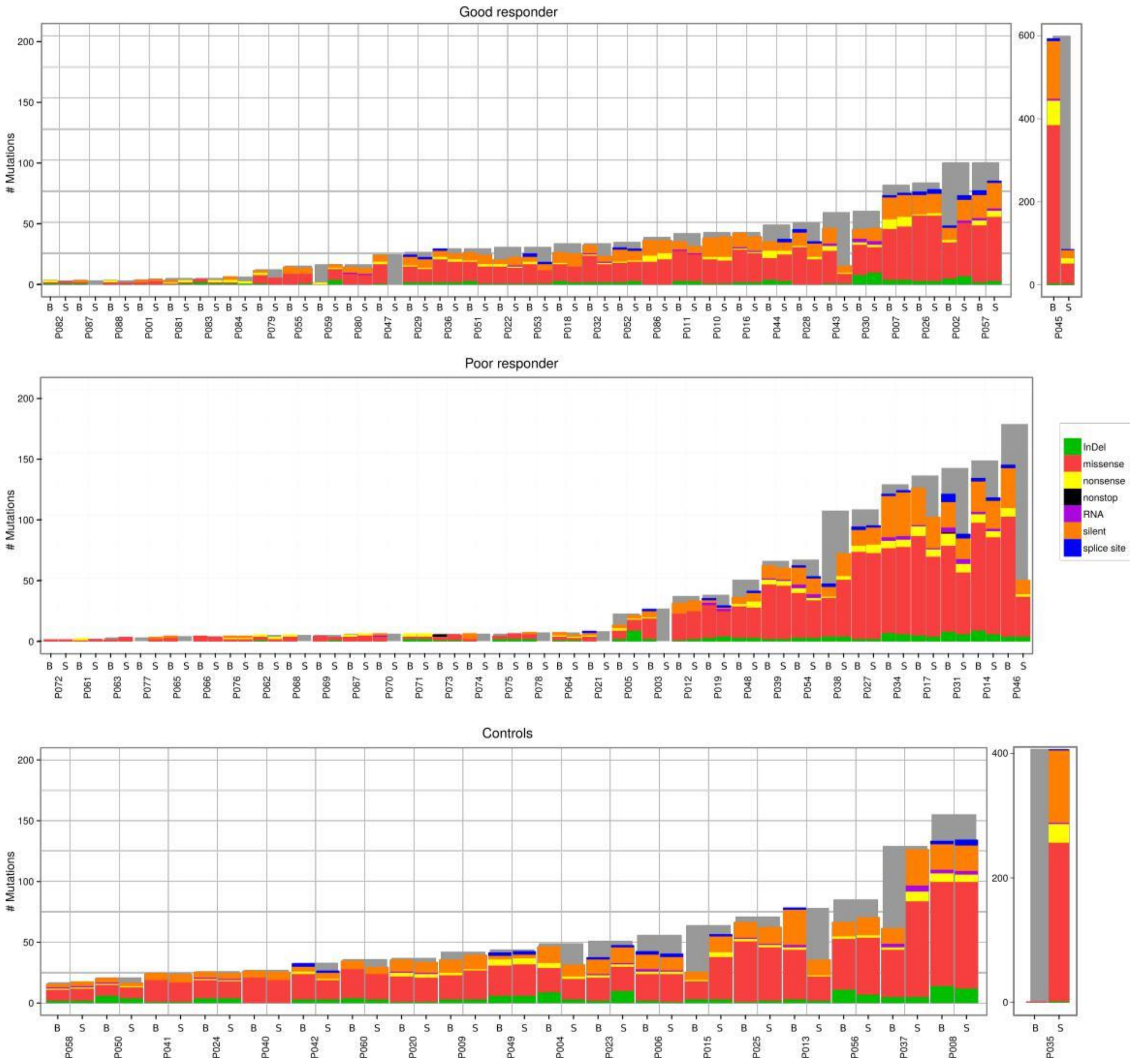

Shown are the number of mutations per sample and their mutation type. Grey bars show the number of mutations per patient by combining the mutations from the baseline (B) and surgery (S) sample. The two outliers (P035 and P045) with much more mutations than other samples are shown on the right at a different scale.

Supplementary Fig. 4: Two samples with highly different mutation count.

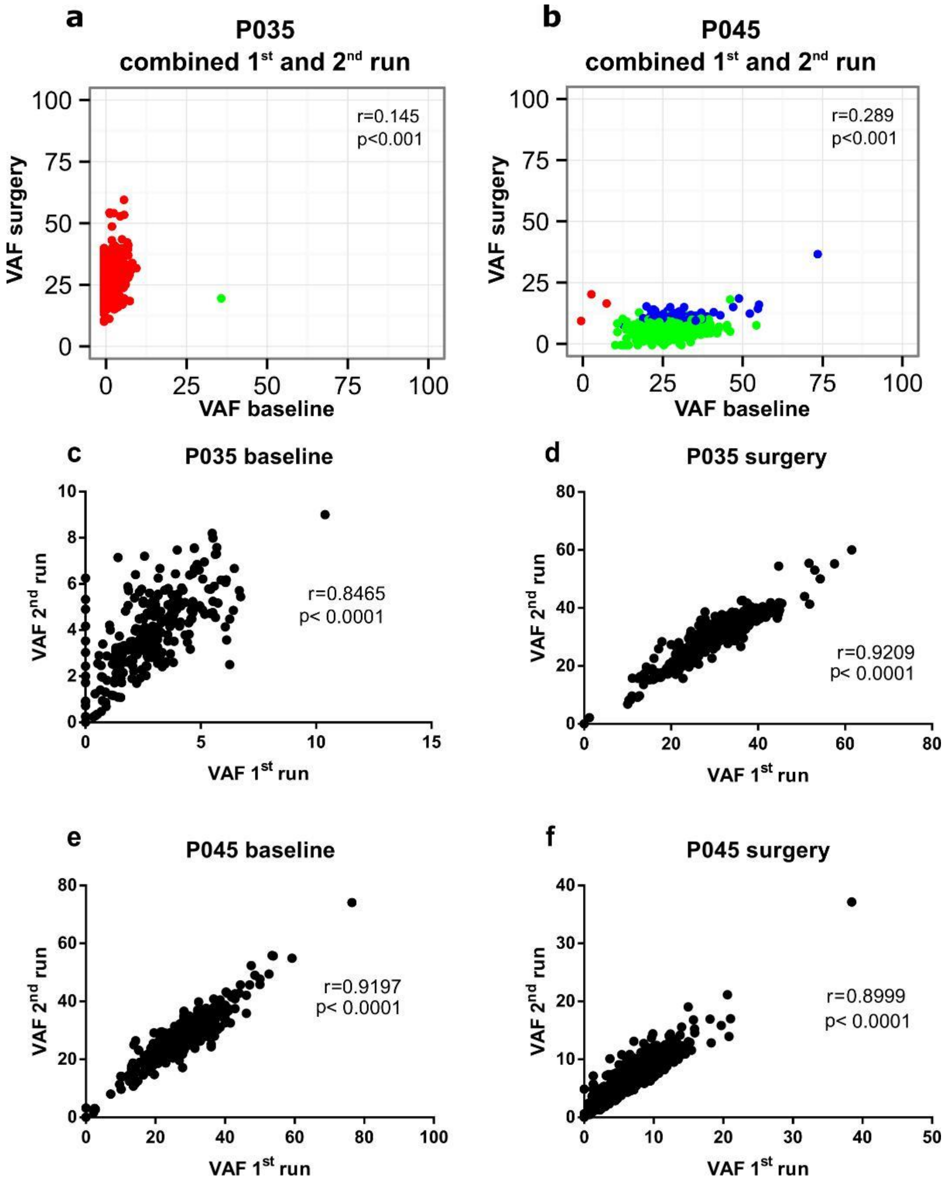

Two samples showed large differences in the mutation count between baseline and surgical sample. Patient P035 had mutations identified almost exclusively in the surgical sample, patient P045 much more in the baseline sample (1st run). To confirm this finding, the samples were sequenced a second time on the capture panel (together with samples from Phase II, 2nd run). On the second run we calculated the variant allele fractions (VAFs) at all positions where a mutation was found in the first run (at baseline and surgical combined)

and found high reproducibility for the samples with more mutations (P035 surgery, r=0.87, **d**; P045 baseline, r=0.88, **e**; Pearson correlation). We also compared the VAFs in the two samples with lower mutation count on mutations identified in the other sample of the pair. Although these mutations had low VAFs and would have not been detected confidently by the variant caller in these samples, strong correlations were found (P035 baseline, r=0.86, **c**; P045 surgery, r=0.87, **f**; Pearson correlation). This shows the robustness and reproducibility of the sequencing approach and suggests that the low mutation count in one sample of these pairs was from normal contamination. By combining reads from both runs (**a** and **b**, VAFs of mutations are shown and coloured if they were called in the baseline (green), surgery (red) or both (blue) samples), over 200x coverage was achieved per sample. At this higher depth the concordance between the sample pairs was still low; only one mutation in P035 was present in B and S, the other 405 mutations only in S. For P045 the majority of the mutations were present in B only (590), few in S only (3) and some in both pairs (83). For P035 the estimated tumour purity was 15% and 70% for B and S, respectively and for P045

the purity is 56% and 23% for B and S, respectively as estimated by using Sequenza[1] based on whole exome sequencing data (see Methods).

Supplementary Fig. 5: Variant allele fractions of paired samples.

Good responder

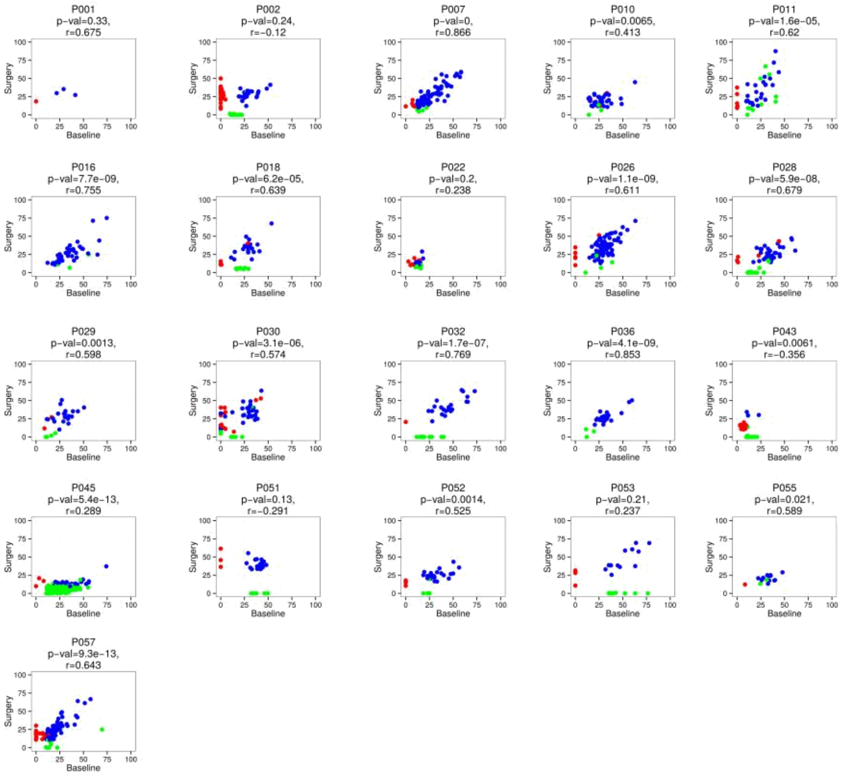

Poor responder

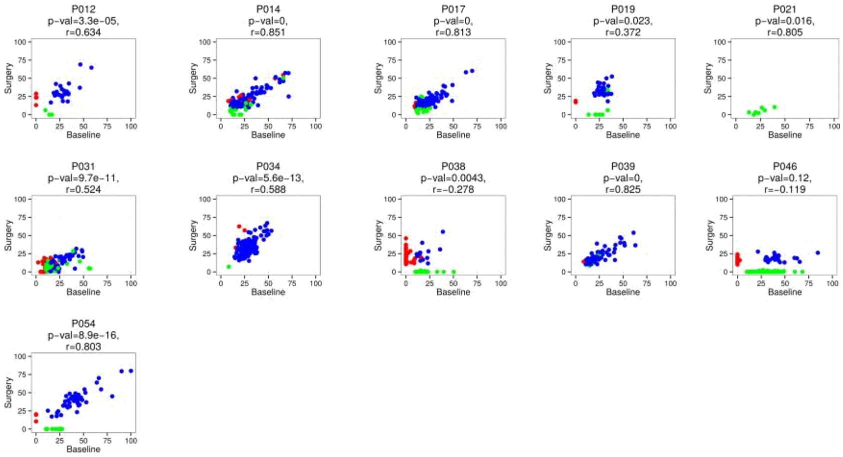

Control

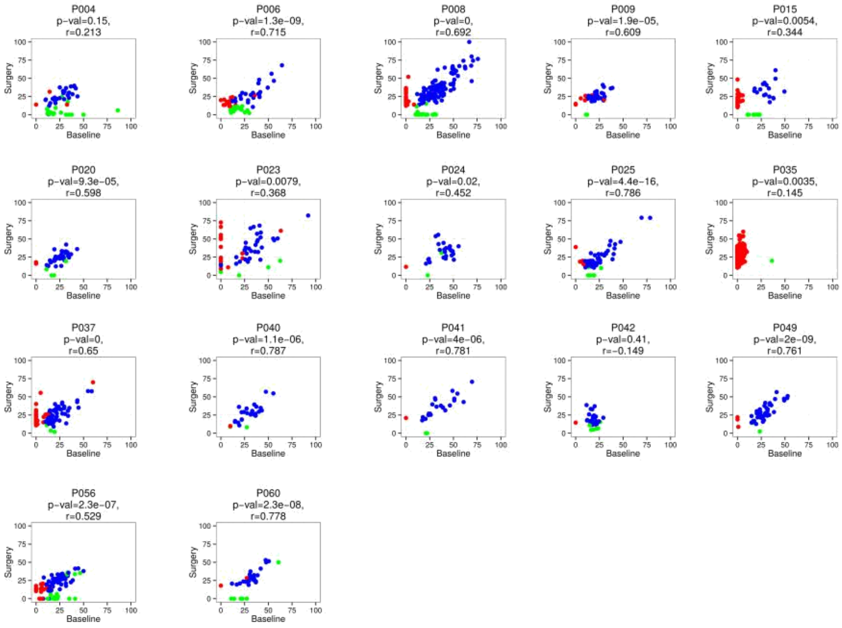

Each plot shows the variant allele fractions (VAFs) of identified mutations in paired samples. Colours indicate if mutations were identified by the somatic variant caller in both samples of the pair (blue), only the baseline sample (green) or only the surgery sample (red). Pearson's correlation coefficient and p-value were calculated based on all mutations.

Supplementary Fig. 6: SciClone plots controls.

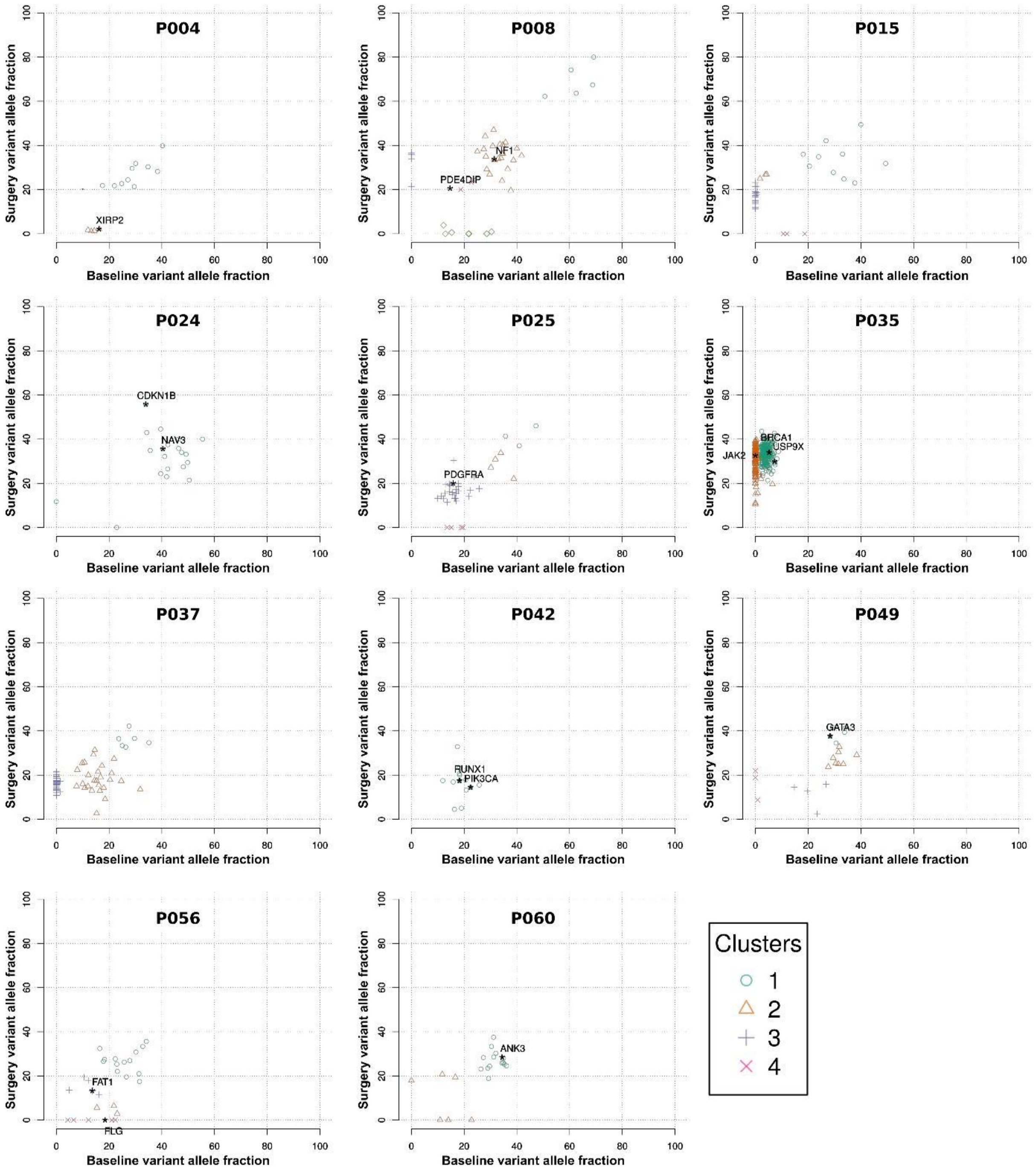

Supplementary Fig. 7: SciClone plots good responders.

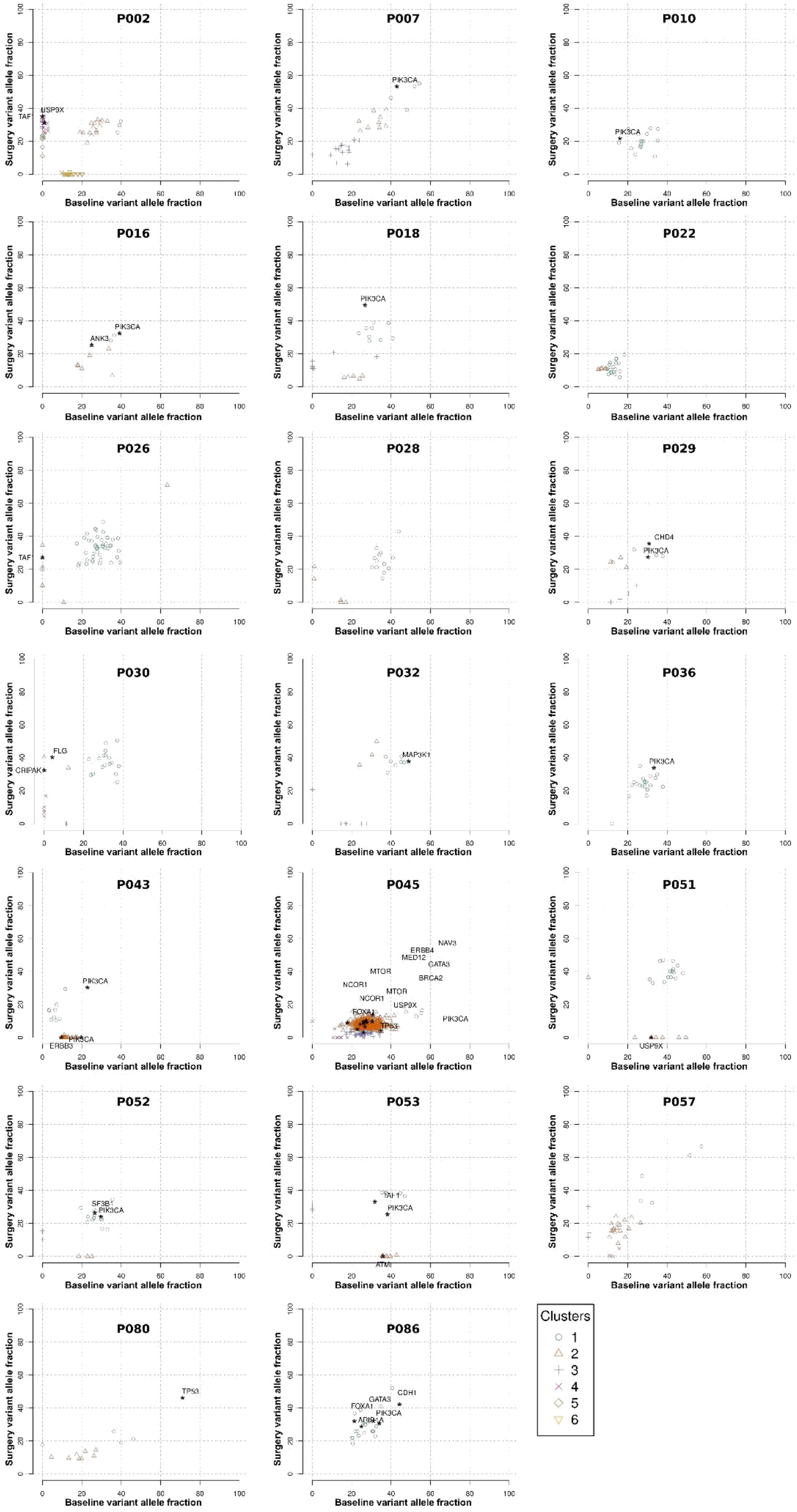
P002
USP9X
TAF
P007
PIK3CA
P010
PIK3CA
P016
PIK3CA
ANK3
P018
PIK3CA
P022
P026
TAF
P028
P029
CHD4
PIK3CA
P030
FLG
CRIPAK
P032
MAP3K1
P036
PIK3CA
P043
PIK3CA
ERBB3
PIK3CA
P045
NAV3
ERBB4
MED12
GATA3
MTOR
BRCA2
NCOR1
MTOR
NCOR1
USP9X
FOXA1
TP53
PIK3CA
P051
USP9X
P052
SF3B1
PIK3CA
P053
TAF1
PIK3CA
ATM
P057
P080
TP53
P086
CDH1
GATA3
FOXA1
PIK3CA
ARID1A
Surgery variant allele fraction
Baseline variant allele fraction
Clusters
1
2
3
4
5
6

Supplementary Fig. 8: SciClone plots poor responders.

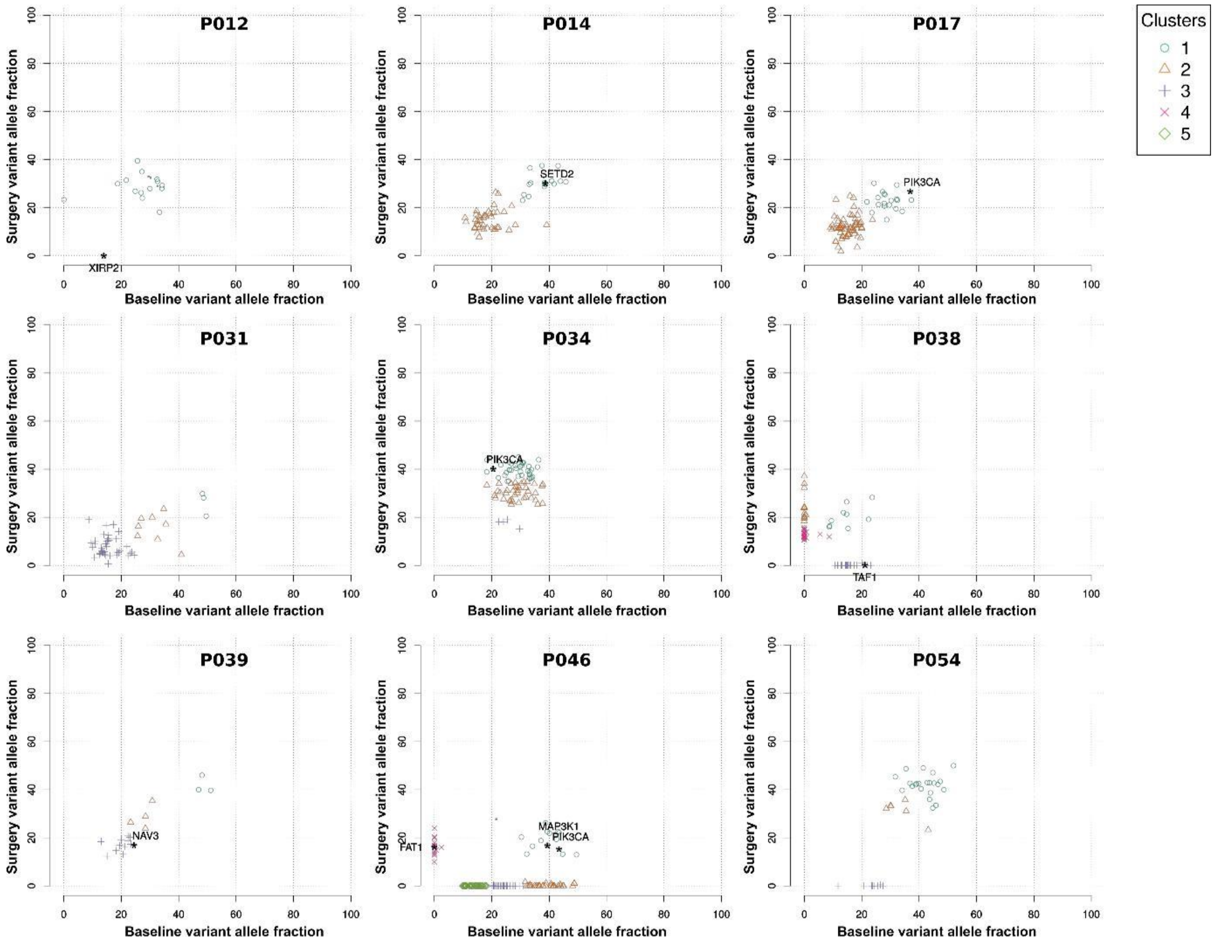

Supplementary Fig. 9: SNP profile.

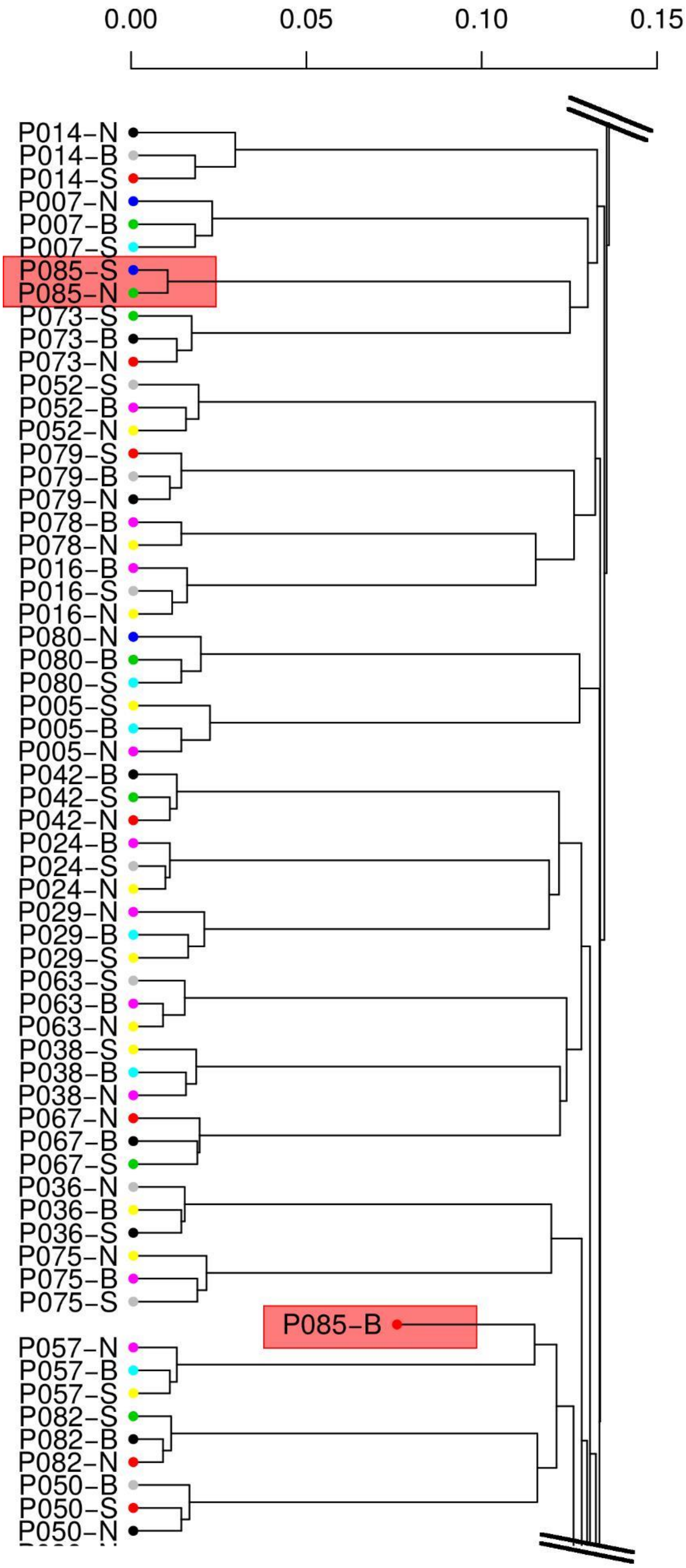

Dendrogram of all samples used in this study based on single-nucleotide polymorphisms (SNPs) from the sequencing data. See Supplementary Material and Methods for details. For all patients the blood, baseline and surgery samples clustered together, except for the baseline sample of patient P085 as highlighted in red. All three samples from this patient were excluded from further analysis. Figure shows an extract of the dendrogram only.

Supplementary Fig. 10: Differences in mutation count between Phase I and Phase II.

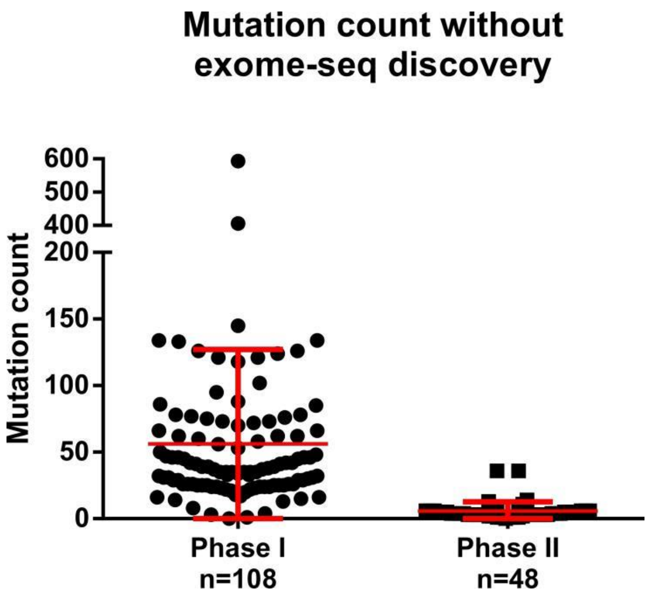

Samples from Phase I underwent whole exome sequencing for somatic mutation discovery. Based on these mutations the capture probe panel was designed. The same panel was used for additional samples in Phase II. As the capture panel was not specifically designed for individual mutations in these, the number of identified mutations was much less. Therefore, only samples with prior whole exome sequencing were used for analysis on the mutational load. Mann-Whitney test, red bars show median and interquartile ranges.

Supplementary Fig. 11: *TP53* mutations status and Ki67 level

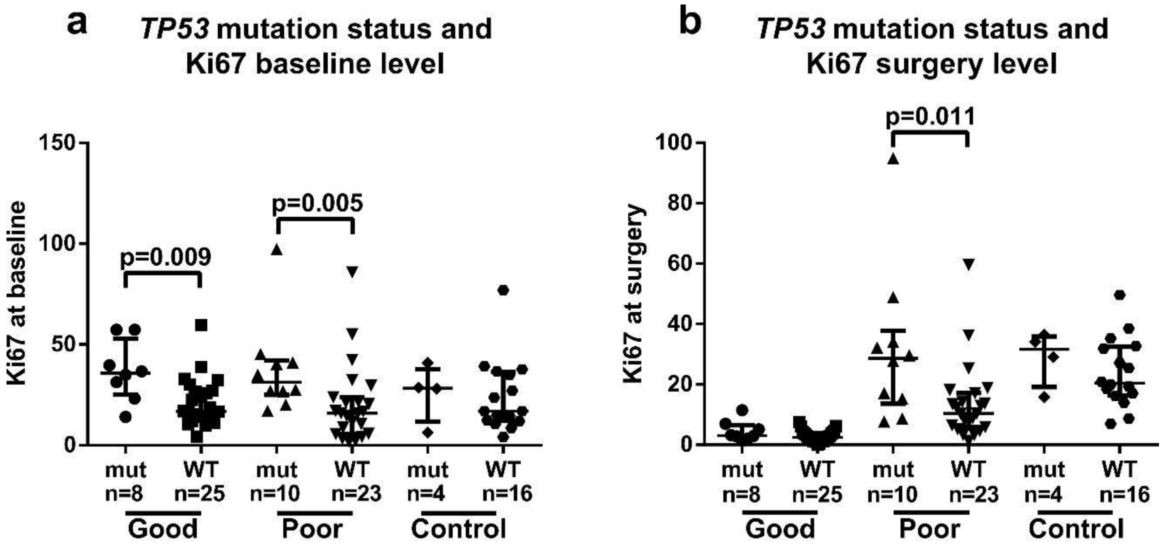

Ki67 levels at baseline (**a**) and surgery (**b**) for patients with mutated *TP53* (mut) and wild-type *TP53* (WT). At baseline good responder and Poor responder had a significantly higher Ki67 level if there was a mutation in *TP53* (good responder median 16.85 vs. 35.81, p=0.009, poor responder median 15.98 vs. 31.26, p=0.005). This difference was lost after treatment for the good responders (median 2.45 vs. 3.05), but not for the poor responders (median 10.34 vs. 28.65, p=0.011). Shown are median and 95% confidence intervals as whiskers, all statistical test were performed with Mann-Whitney.

Supplementary Fig. 12: Cellularity based on field counts

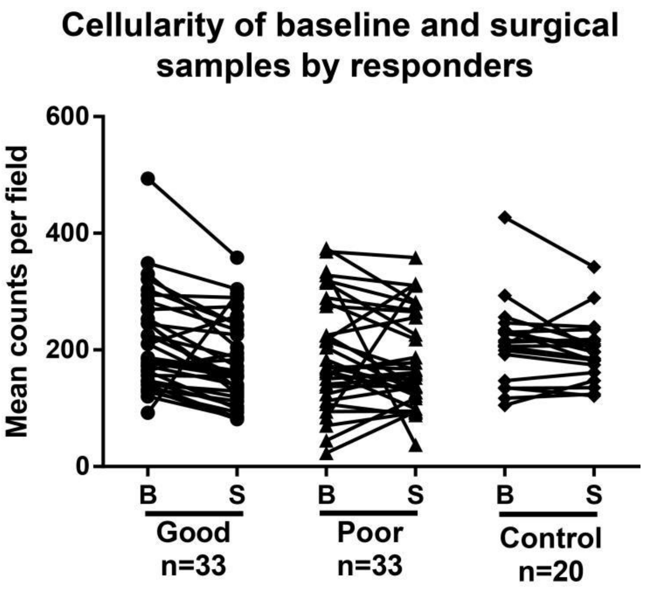

The Good responders showed a significantly lower cellularity in the surgery (S) samples compared to baseline (B) based on field counts (median B 186.8 *vs.* S 157.0, p-value<0.001, Wilcoxon matched-pairs signed rank test). There was no significant difference between the B and S samples for the Poor responders and Controls.

Supplementary Fig. 13: Biopsy types

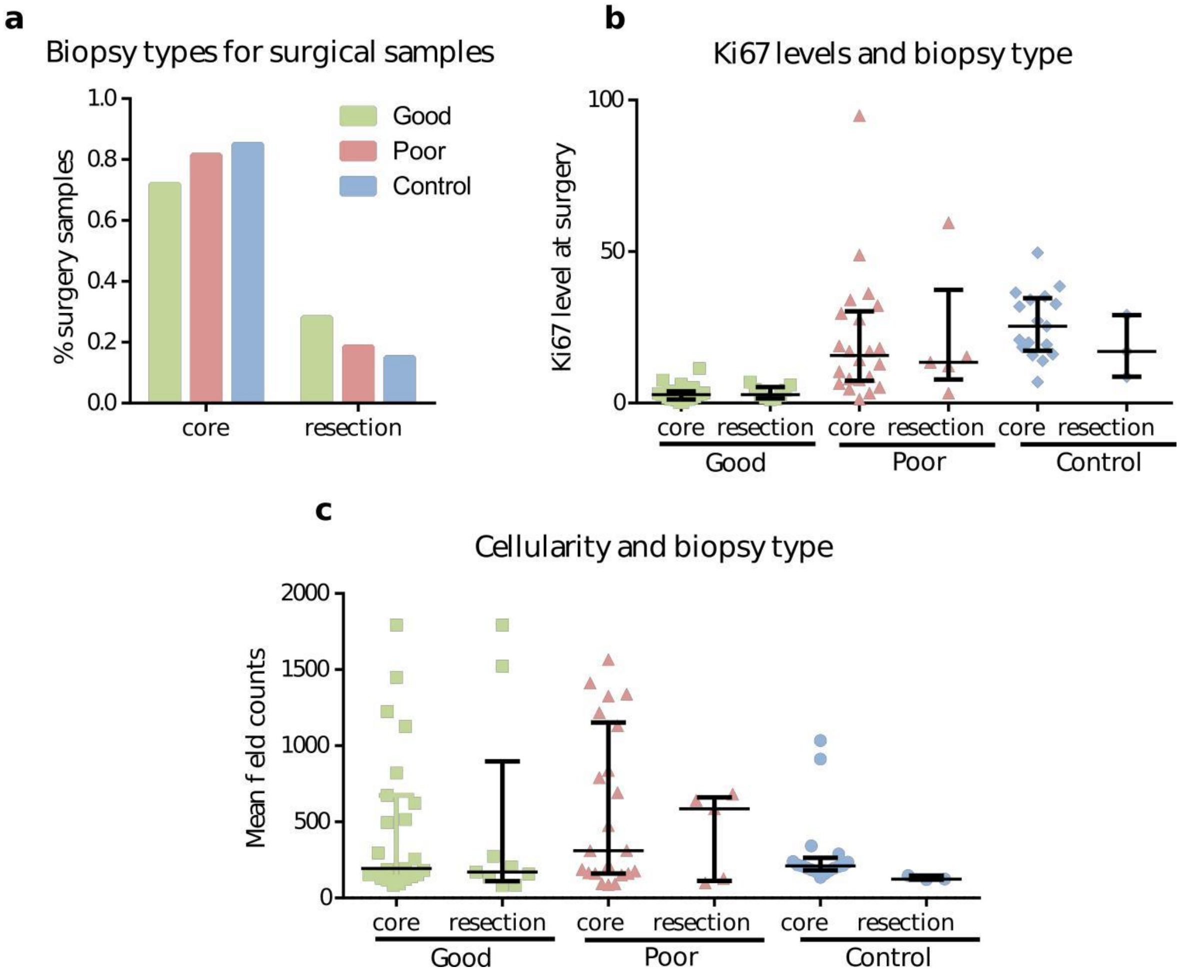

Surgery samples were from core-cuts and resections. (**a**) There was no statistical significant difference in the number of resections across the responder groups (Good n=9, Poor n=5, Control n=3). There was also no difference in the (**b**) Ki67 levels and (**c**) cellularity (based on field counts) in relation to the type of biopsy except for the Control group where a slightly lower cellularity was found in the three samples from resection (Mann-Whitney test, bars show median and interquartile ranges).

Supplementary Tables

Supplementary Table 1: Clinical data summary of 60 patients with whole exome sequencing.

Patient demographics of those patients with whole exome sequencing from Phase I separated by poor responder, good responder and Control.

	**Response group**					
	**Poor (n=15)**		**Good (n=25)**		**Control (n=20)**	
	**n**	**%**	**n**	**%**	**n**	**%**
**PgR status**						
Positive	10	66.7	21	84.0	15	75.0
Negative	3	20.0	3	12.0	3	15.0
Not known	2	13.3	1	4.0	2	10.0
**Histological subtype**						
Ductal	13	86.7	19	76.0	16	80.0
Lobular	1	6.7	3	12.0	2	10.0
Mucinous	0	0.0	1	4.0	0	0.0
Mixed ductal and lobular	1	6.7	0	0.0	1	5.0
Not known	0	0.0	2	8.0	1	5.0
**Pre-treatment tumour grade**						
G1	1	6.7	0	0.0	3	15.0
G2	6	40.0	16	64.0	8	40.0
G3	6	40.0	2	8.0	5	25.0
Not known	2	13.3	7	28.0	3	15.0
Missing	0	0.0	0	0.0	1	5.0

**No. of involved lymph nodes**						
N0	7	46.7	14	56.0	11	55.0
N1-3	5	33.3	9	36.0	6	30.0
N4+	3	20.0	2	8.0	3	15.0
**HER2 status**						
Negative	10	66.7	24	96.0	13	65.0
Positive	5	33.3	1	4.0	7	35.0
**Pre-treatment tumour size (cm)**						
<2	6	40.0	10	40.0	7	35.0
2-5	8	53.3	15	60.0	12	60.0
>5	1	6.7	0	0.0	1	5.0
**Surgery tumour size (cm)**						
<2	3	20.0	8	32.0	8	40.0
2-5	12	80.0	17	68.0	10	50.0
>5	0	0.0	0	0.0	2	10.0
	**Median**	**IQR**	**Median**	**IQR**	**Median**	**IQR**
**Age at randomization (years)**	66	61 - 75	74	63 - 82	70	59 - 76
**Time from randomization to surgery (days)**	19	13 - 23	17	15 - 19	18.5	14 -

Supplementary Table 2: List of 77 breast cancer related genes

In addition to the regions identified as mutated through exome-sequencing, we added all exons of 77 genes of interest manually curated from COSMIC and Ellis *et al*[2] and Piccart et al[3].

ABCA13	*DNAH5*	*LDLRAP1*	*PTPRD*
AGTR2	*DNAH9*	*LRP2*	*RB1*
AKT1	*DNMT3A*	*LYN*	*RELN*
AKT2	*DSPP*	*MACF1*	*RUNX1*
APOB	*ERBB2*	*MAP2K4*	*RYR1*
ARID1A	*ESR1*	*MAP3K1*	*RYR2*
ATM	*FAT3*	*MDN1*	*RYR3*
ATR	*FCGBP*	*MED12*	*SF3B1*
BAP1	*FGFR1*	*MLL3*	*SPEN*
BRAF	*FGFR2*	*MYH9*	*SPTA1*
BRCA2	*FGFR3*	*NCOA3*	*STMN2*
CBFB	*FLG*	*NCOR1*	*SYNE1*
CCND1	*FRG1B*	*NCOR2*	*SYNE2*
CDH1	*GATA3*	*NEB*	*TBX3*
CDKN1B	*GOLGA6L2*	*NF1*	*TP53*
CSMD1	*HMCN1*	*OBSCN*	*USH2A*
CSMD2	*HRNR*	*PIK3CA*	*ZFHX4*
DMD	*HUWE1*	*PIK3R1*	
DNAH11	*IGF1R*	*PKHD1L1*	
DNAH3	*KRAS*	*PTEN*	

Supplementary Table 3: Frequently mutated genes.

Genes that were mutated in 10% or more of the 86 patients in this study are listed. As comparison the percentage of patients with mutations in TCGA are shown. Only ER+ breast cancers from post-menopausal patients were selected from TCGA. Apart from the genes shown at the top of the table, only three other genes (*GATA3*, *RYR2* and *MAP3K1*) were mutated with a frequency >5% in tumours from TCGA. These genes were mutated at a lower, but similar frequency in our data set.

Gene	Patients with mutations	% Patients with mutations	% ER+ tumours with mutations in TCGA
PIK3CA	32	37%	35%
TP53	22	26%	18%
CDH1	12	14%	15%
MLL3	12	14%	8%
ABCA13	10	12%	4%
FLG	9	10%	5%
GATA3	6	7%	9%
RYR2	5	6%	6%
MAP3K1	4	5%	9%

Supplementary Table 4: Concordance with driver gene mutations from DriverDB.

Mutations found in 94 breast cancer driver genes according to DriverDB for the 77 sample pairs. Mutations identical in baseline and surgery were counted separately and the percentage of identical mutations is shown.

Patient ID	Driver genes mutated in baseline/surgery sample	Identical mutations between baseline and	% identical
P001	1/1	1	100%
P002	1/3	1	33%
P004	3/3	3	100%
P005	3/4	3	75%
P006	5/4	4	80%
P007	3/3	3	100%
P008	4/4	3	60%
P009	0/0	0	0%
P010	1/2	1	50%
P011	1/1	1	100%
P012	0/0	0	0%
P013	3/0	0	0%
P014	4/3	3	75%
P015	3/4	2	40%
P016	3/3	3	100%
P017	3/2	2	67%
P018	2/1	1	50%
P019	0/0	0	0%
P020	0/0	0	0%
P021	1/0	0	0%
P022	1/1	1	100%
P023	1/2	1	50%
P024	0/0	0	0%
P025	4/4	4	100%
P026	1/1	1	100%
P027	2/2	2	100%
P028	1/1	1	100%
P029	3/3	3	100%
P030	2/3	2	67%
P031	4/3	2	40%
P032	3/3	3	100%
P034	4/5	4	80%
P035	0/13	0	0%
P036	2/1	1	50%
P037	4/5	4	80%

P038	4/4	2	33%
P039	1/1	1	100%
P040	0/0	0	0%
P041	0/0	0	0%
P042	4/3	3	75%
P043	3/2	1	25%
P044	1/1	1	100%
P045	22/2	2	9%
P046	7/7	4	40%
P048	4/2	2	50%
P049	2/3	2	67%
P050	3/3	2	50%
P051	0/1	0	0%
P052	2/2	2	100%
P053	2/1	1	50%
P054	2/2	2	100%
P055	0/0	0	0%
P056	7/4	4	57%
P061	2/1	1	50%
P057	1/2	1	50%
P058	0/0	0	0%
P062	3/1	1	33%
P079	5/3	3	60%
P063	0/0	0	0%
P064	2/2	2	100%
P080	1/1	1	100%
P081	1/2	1	50%
P082	2/1	1	50%
P083	0/0	0	0%
P060	2/2	2	100%
P084	3/2	2	67%
P066	1/1	1	100%
P067	2/2	2	100%
P086	10/11	10	91%
P087	1/0	0	0%
P069	0/0	0	0%
P088	1/0	0	0%
P071	3/3	3	100%
P072	1/1	1	100%
P073	3/2	2	67%
P075	2/2	2	100%
P076	2/2	2	100%

Supplementary Table 5: Histologies per patient.

Availability of sequencing data along with Ki67 measurements for each patient in this study. Treated patients were classified as poor responders with a Ki67 reduction of <60% between baseline and surgery and as good responders with >75% Ki67 reduction. Controls did not receive treatment and were not selected for Ki67 change. HER2 status of the surgical sample as measured by immunohistochemistry (IHC) or fluorescence *in situ* hybridization (FISH) is shown.

ID	Group	Paired Exome-Sequencing available	Paired targeted sequencing	Her2 IHC/FISH	Ki67 baseline	Ki67 surgery	Ki67 % change
P001	Good	paired	paired	negative	25.98	4.54	-82.51
P002	Good	paired	paired	negative	14.45	0.12	-99.18
P003	Poor	pre	pre	negative	15.98	8.75	-45.25
P004	Control	paired	paired	negative	10.5	8.68	-17.32
P005	Poor	post	paired	negative	8.8	6.46	-26.58
P006	Control	pre	paired	positive	40.79	34.1	-16.4
P007	Good	paired	paired	negative	16.95	3.91	-76.95
P008	Control	paired	paired	positive	14.94	25.36	69.79
P009	Control	paired	paired	negative	11.92	31.84	167.09
P010	Good	paired	paired	negative	14.59	1.6	-89.03
P011	Good	paired	paired	negative	18.64	1.77	-90.5
P012	Poor	paired	paired	positive	42.36	17.15	-59.51
P013	Control	paired	paired	positive	76.92	49.59	-35.54
P014	Poor	paired	paired	negative	97.31	94.86	-2.52
P015	Control	paired	paired	positive	6.34	15.83	149.61
P016	Good	paired	paired	negative	38.91	7.54	-80.63
P017	Poor	paired	paired	negative	45.19	29.62	-34.46
P018	Good	paired	paired	negative	27.07	4.2	-84.49
P019	Poor	paired	paired	negative	20.44	10.34	-49.43
P020	Control	paired	paired	positive	16.99	13.93	-18.03
P021	Poor	paired	paired	negative	20	8.69	-56.56

P022	Good	paired	paired	positive	39.63	3.24	-91.82
P023	Control	paired	paired	negative	34.9	32.68	-6.35
P024	Control	paired	paired	negative	14.61	19.96	36.62
P025	Control	paired	paired	positive	37.72	19.25	-48.98
P026	Good	paired	paired	negative	32.97	1.17	-96.45
P027	Poor	post	paired	positive	85.8	36.21	-57.8
P028	Good	paired	paired	negative	16.85	3.61	-78.58
P029	Good	paired	paired	negative	36.72	2.07	-94.36
P030	Good	paired	paired	negative	16.49	2.72	-86.04
P031	Poor	paired	paired	negative	34.95	34.08	-2.49
P032	Good	paired	paired	negative	32.13	6.23	-80.61
P078	Poor	none	pre	positive	21.55	13.69	-36.48
P034	Poor	paired	paired	negative	23.81	13.48	-43.38
P035	Control	paired	paired	negative	8.62	16.16	87.47
P036	Good	paired	paired	negative	13.33	2.45	-81.62
P037	Control	paired	paired	negative	28.5	36.42	27.79
P038	Poor	paired	paired	positive	41.1	27.67	-32.68
P039	Poor	paired	paired	positive	27.57	15.25	-44.69
P040	Control	paired	paired	positive	39.22	38.55	-1.71
P041	Control	paired	paired	negative	27.11	35.21	29.88
P042	Control	paired	paired	negative	16.98	17.07	0.13
P043	Good	paired	paired	negative	14.09	2.86	-79.7
P044	Good	post	paired	negative	59.65	2.77	-95.36
P045	Good	paired	paired	negative	23.19	2.77	-88.04
P046	Poor	paired	paired	negative	10.54	4.6	-56.36
P047	Good	pre	pre	negative	15.47	1.63	-89.48
P048	Poor	post	paired	positive	26.93	17.14	-36.38
P049	Control	paired	paired	negative	36.7	27.09	-26.19
P050	Control	post	paired	negative	4.09	6.94	69.58
P051	Good	paired	paired	negative	9.7	1.08	-88.82
P052	Good	paired	paired	negative	11.15	1.29	-88.43
P053	Good	paired	paired	negative	14.23	1.82	-87.21
P054	Poor	paired	paired	negative	26.84	32.18	19.92
P055	Good	paired	paired	negative	4.26	0.22	-94.87
P056	Control	paired	paired	negative	28.03	29.08	3.75

P061	Poor	none	paired	negative	2.33	1.26	-45.92
P057	Good	paired	paired	negative	57.31	6.92	-87.93
P058	Control	post	paired	negative	12.42	18.46	48.64
P062	Poor	none	paired	positive	17	7.71	-54.65
P079	Good	none	paired	negative	15.32	3.38	-77.94
P063	Poor	none	paired	negative	3.28	12.31	275.3
P064	Poor	none	paired	negative	32.32	18.84	-41.71
P059	Good	none	post	negative	34.9	5.1	-85.39
P080	Good	none	paired	negative	57.35	11.42	-80.09
P081	Good	none	paired	negative	22.51	1.23	-94.54
P082	Good	none	paired	negative	30.19	6.08	-79.86
P083	Good	none	paired	negative	12.04	1.24	-89.7
P060	Control	paired	paired	negative	23.63	20.91	-11.51
P084	Good	none	paired	negative	31.37	1.41	-95.5
P065	Poor	none	pre	positive	55.02	25.33	-53.96
P066	Poor	none	paired	negative	17	59.5	3.5
P067	Poor	none	paired	negative	29.51	18.11	-38.63
P086	Good	none	paired	negative	10.38	1.12	-89.21
P087	Good	none	paired	negative	24.14	3.99	-83.47
P068	Poor	none	pre	negative	15.81	7.98	-49.53
P069	Poor	none	paired	negative	4.18	5.19	24.16
P088	Good	none	paired	negative	26.51	2.96	-88.83
P070	Poor	none	pre	negative	1.79	5.88	228.49
P071	Poor	none	paired	negative	40.33	48.86	21.15
P072	Poor	none	paired	negative	5.52	3.32	-39.86
P073	Poor	none	paired	negative	5.69	3.32	-39.86
P074	Poor	none	pre	negative	11.68	10.29	-11.9
P075	Poor	none	paired	negative	14.66	8.07	-44.95
P076	Poor	none	paired	negative	21.25	14.31	-32.66
P077	Poor	none	post	negative	16.78	12.91	-23.06

Supplementary Table 6: Extended list of cancer related genes.

List of frequently mutated genes in cancer derived from literature. The MAF files were obtained from supplementary files[4-8] associated with each publication and were merged into a single list containing 65,880 mutations observed in one or more of 1,273 patients. The list is then filtered for non-silent mutations and genes mutated in at least 1% of the patients.

ABCA13	*DNAH5*	*LYN*	*PTPRB*
ADAMTS20	*DNAH6*	*LYST*	*PTPRD*
ADAMTSL1	*DNAH7*	*MACF1*	*QSER1*
ADCY9	*DNAH8*	*MADD*	*RB1*
AFF2	*DNAH9*	*MALAT1*	*RBMX*
AGTR2	*DOCK11*	*MAP1A*	*RELN*
AHNAK	*DSP*	*MAP2*	*RIMS2*
AHNAK2	*DSPP*	*MAP2K4*	*RP1*
AKAP9	*DST*	*MAP3K1*	*RPGR*
AKD1	*DYNC1H1*	*MAP3K4*	*RUNX1*
AKT1	*DYNC2H1*	*MDM2*	*RYR1*
ANK1	*ERBB2*	*MDM4*	*RYR2*
ANK3	*ERBB3*	*MDN1*	*RYR3*
ANKRD12	*ERBB4*	*MED12*	*SCN10A*
ANKRD30A	*ESR1*	*MED23*	*SCN1A*
AOAH	*EYS*	*MEF2A*	*SCN2A*
APOB	*F5*	*MET*	*SCN3A*
ARID1A	*FAM135B*	*MGA*	*SCN5A*
ARID1B	*FAM157B*	*MGAM*	*SCN7A*
ARID2	*FAM157C*	*MLL*	*SDK1*
ASPM	*FAM171A1*	*MLL2*	*SDK2*
ASXL3	*FAM186A*	*MLLT4*	*SETD2*
ATM	*FAM47C*	*MST1P9*	*SETX*
ATN1	*FAT1*	*MT-CYB*	*SF3B1*
ATP10B	*FAT3*	*MTOR*	*SHROOM4*
ATR	*FAT4*	*MXRA5*	*SI*
BAT2L1	*FBN1*	*MYB*	*SMG1*
BIRC6	*FBN3*	*MYCBP2*	*SPEN*
BRAF	*FCGBP*	*MYH11*	*SPHKAP*
BRCA1	*FER1L5*	*MYH14*	*SPI1*
BRCA2	*FLG*	*MYH6*	*SPTA1*
BRWD3	*FLG2*	*MYH8*	*SPTB*
C10orf18	*FLNB*	*MYH9*	*SRCAP*
C12orf51	*FMN2*	*MYO3A*	*SRRM2*
C5orf42	*FOXA1*	*MYO5B*	*STAB2*
CACNA1A	*FRG1B*	*MYO7A*	*SVEP1*

CACNA1B	*FRY*	*MYO9A*	*SYCP2*
CACNA1C	*GATA3*	*MYST4*	*SYNE1*
CACNA1E	*GIGYF2*	*NAV3*	*SYNE2*
CACNA1F	*GOLGB1*	*NBAS*	*TAF1*
CASP8	*GON4L*	*NBEAL2*	*TAF1L*
CASR	*GPR112*	*NBPF1*	*TBX3*
CBFB	*GPR98*	*NBPF10*	*TEP1*
CCDC66	*GRIN2A*	*NCOA3*	*TEX15*
CD163L1	*HCFC1*	*NCOR1*	*TG*
CDC42BPA	*HEATR1*	*NCOR2*	*TLN1*
CDH1	*HECTD1*	*NEB*	*TLR4*
CDKN1B	*HECW1*	*NF1*	*TNXB*
CENPE	*HECW2*	*NHS*	*TP53*
CEP350	*HERC1*	*NOTCH4*	*TPR*
CFH	*HERC2*	*NR1H2*	*UBR4*
CHD4	*HGC6.3*	*NUP160*	*UBR5*
CHD6	*HMCN1*	*OBSCN*	*UNC5D*
CIT	*HRNR*	*ODZ1*	*USH2A*
CMYA5	*HUWE1*	*ODZ4*	*USP32*
CNTLN	*HYDIN*	*OTOF*	*USP34*
COL12A1	*INSRR*	*PCDH15*	*USP36*
COL6A3	*ITPR1*	*PCDH19*	*USP9X*
COL6A6	*JAK1*	*PCLO*	*UTRN*
COL7A1	*JAK2*	*PCNT*	*VPS13C*
CRIPAK	*KIF26B*	*PCNXL2*	*VPS13D*
CROCCL1	*KIT*	*PCSK5*	*VWF*
CSF1R	*KRAS*	*PDE4DIP*	*WDFY3*
CSMD1	*LAMA1*	*PDGFRA*	*XIRP2*
CSMD2	*LAMA2*	*PDZD2*	*ZDBF2*
CSMD3	*LAMA3*	*PHKA2*	*ZFHX3*
CTCF	*LAMB4*	*PIK3CA*	*ZFHX4*
CUBN	*LAMC1*	*PIK3R1*	*ZFPM2*
DCC	*LOC283685*	*PKD1L1*	*ZNF384*
DCHS1	*LOC440300*	*PKHD1*	*ZNF462*
DCHS2	*LOC442421*	*PKHD1L1*	
DDR1	*LOC642236*	*PLCE1*	
DIDO1	*LOC643677*	*PLXNA4*	
DLC1	*LOC646214*	*POLE*	
DMD	*LOC729057*	*POLQ*	
DNAH10	*LRBA*	*PREX1*	
DNAH11	*LRP1*	*PREX2*	
DNAH17	*LRP1B*	*PRKDC*	
DNAH2	*LRP2*	*PRUNE2*	
DNAH3	*LTK*	*PTEN*	

Supplementary References

1 Favero, F. *et al.* Sequenza: allele-specific copy number and mutation profiles from tumor sequencing data. *Ann Oncol* **26**, 64-70, doi:10.1093/annonc/mdu479 (2015).

2 Ellis, M. J. *et al.* Whole-genome analysis informs breast cancer response to aromatase inhibition. *Nature* **486**, 353-360, doi:10.1038/nature11143 (2012).

3 Piccart, M. *et al.* Assesment of genetic alterations in postmenopausal women with hormone receptor-positive, HER2-negative advanced breast cancer from the BOLERO-2 trail by next generation sequencing. *Ann. Oncol.* **24**, iii25-iii26 (2013).

4 Banerji, S. *et al.* Sequence analysis of mutations and translocations across breast cancer subtypes. *Nature* **486**, 405-409, doi:10.1038/nature11154 (2012).

5 Cancer Genome Atlas, N. Comprehensive molecular portraits of human breast tumours. *Nature* **490**, 61-70, doi:10.1038/nature11412 (2012).

6 Shah, S. P. *et al.* The clonal and mutational evolution spectrum of primary triple-negative breast cancers. *Nature* **486**, 395-399, doi:10.1038/nature10933 (2012).

7 Stephens, P. J. *et al.* The landscape of cancer genes and mutational processes in breast cancer. *Nature* **486**, 400-404, doi:10.1038/nature11017 (2012).

8 Kan, Z. *et al.* Diverse somatic mutation patterns and pathway alterations in human cancers. *Nature* **466**, 869-873, doi:10.1038/nature09208 (2010).
